# Reversible chromatin condensation by the DNA repair and demethylation factor thymine DNA glycosylase

**DOI:** 10.1093/nar/gkab040

**Published:** 2021-02-08

**Authors:** Charles E Deckard, Jonathan T Sczepanski

**Affiliations:** Department of Chemistry, Texas A&M University, College Station, TX 77843, USA; Department of Chemistry, Texas A&M University, College Station, TX 77843, USA

## Abstract

Chromatin structures (and modulators thereof) play a central role in genome organization and function. Herein, we report that thymine DNA glycosylase (TDG), an essential enzyme involved in DNA repair and demethylation, has the capacity to alter chromatin structure directly through its physical interactions with DNA. Using chemically defined nucleosome arrays, we demonstrate that TDG induces decompaction of individual chromatin fibers upon binding and promotes self-association of nucleosome arrays into higher-order oligomeric structures (i.e. condensation). Chromatin condensation is mediated by TDG’s disordered polycationic N-terminal domain, whereas its C-terminal domain antagonizes this process. Furthermore, we demonstrate that TDG-mediated chromatin condensation is reversible by growth arrest and DNA damage 45 alpha (GADD45a), implying that TDG cooperates with its binding partners to dynamically control chromatin architecture. Finally, we show that chromatin condensation by TDG is sensitive to the methylation status of the underlying DNA. This new paradigm for TDG has specific implications for associated processes, such as DNA repair, DNA demethylation, and transcription, and general implications for the role of DNA modification ‘readers’ in controlling chromatin organization.

## INTRODUCTION

Eukaryotic genomes are hierarchically organized into a nucleoprotein complex called chromatin. Nucleosomes, the basic unit of chromatin, interact with each other over short distances to form locally compact structures (e.g. 30 nm diameter fibers) that modulate DNA accessibility at the level of single genes (1). On a larger scale, long-range chromatin fiber contacts within and between chromosomes drive the condensation of chromatin into distinct structural domains that are key to genome organization and function (2). Unraveling the molecular mechanisms underlying the formation and regulation of these locally and globally condensed chromatin structures, and identifying the protein factors involved, is essential for understanding the fundamental genomic processes of the cell.

Thymine DNA glycosylase (TDG) was originally described as a DNA repair enzyme capable of excising pyrimidine bases from G•T pairs that arise from 5-methylcytosine (5mC) deamination (3,4). However, it is now clear that TDG’s role in biology extends well beyond DNA repair. As the only known enzyme capable of removing 5-formylcytosine (5fC) and 5-carboxycytosine (5caC) from DNA in mammals (5,6), TDG plays an essential role in controlling DNA methylation dynamics (7,8). In addition to its catalytic roles, TDG has been shown to function as a transcriptional co-activator through its association with various transcription factors and activating histone modifiers (9,10), such as the histone acetyltransferases CBP and p300 (11), thereby coordinating the formation of a transcriptionally permissive chromatin state. TDG also mediates long-range physical contacts between promoters and enhancers at a subset of hormone responsive genes (12,13). Interestingly, some TDG-bound enhancers are incorporated into phase-separated transcriptional compartments along with other TDG-interacting proteins, further connecting TDG with chromatin architecture (14). While TDG’s role in chromatin organization is generally viewed as indirect, we recognized that TDG possesses several features that suggest it may have the capacity to alter chromatin structure *directly*: TDG binds non-specifically to DNA (15) and nucleosomes (16) *in vitro* and contains an intrinsically disordered lysine-rich regulatory domain that closely resembles the C-terminus of linker histone H1, a basic peptide known to promote chromatin fiber folding and condensation (17–19). Herein, we now provide the first experimental evidence that TDG can directly alter chromatin structure through its physical interactions with DNA. Importantly, we show that TDG promotes condensation of chromatin fibers into higher-order oligomeric structures, thereby linking TDG-dependent pathways to long-range chromatin organization.

## MATERIALS AND METHODS

### Reagents

Restriction enzymes (EcoRV, PflMI, BstXI, DraIII-HF, HaeII, DraI), UDG, hSMUG, NEB Next dsDNA Fragmentase, M.SssI CpG Methyltransferase, and micrococcal nuclease (MNase) were purchased from New England Biolabs (Ipswich, MA). Maleimide-Cy3 and Cy5 dyes (cat. nos. 21380, 23380) used in the labeling of H2A_N110C_ were acquired from Lumiprobe Life Science Solutions (Hunt Valley, MD, USA). Sequencing grade trypsin (cat. no. 90057) was purchased from Fisher Scientific (Waltham, MA, USA). Synthetic oligonucleotides were either purchased from Integrated DNA Technologies (IDT) or prepared by solid-phase synthesis on an Expedite 8909 DNA/RNA synthesizer using standard methods. DNA synthesis reagents and nucleoside phosphoramidites were purchased from Glen Research (Sterling, VA, USA). Mixed human genomic DNA (cat. no. G3041) was purchased from Promega Corp. (Madison, WI, USA). Tail-less histone proteins (H3 residues 38–135, H4 residues 17–99) were purchased from the Histone Source (Ft. Collins, CO, USA). Histone H1.1 (cat. no. ab198676) was purchased from Abcam (Cambridge, MA, USA).

### Methods

#### Histone preparation and octamer refolding

Recombinant human histones (H2A_N1110C_, H2A.1, H2B.1, H3.1 and H4.1) were expressed and purified using established protocols (20,21). Histone H2A_N1110C_ was fluorescently labeled with maleimide Cy3 and Cy5 dyes using an established protocol (22), and histone octamers were refolded and purified as previously described (20,21). Purified histone octamers were stored at 4°C in Octamer Buffer (2 M NaCl, 5 mM BME, 0.2 mM PMSF, 10 mM HEPES, pH 7.8) until further use.

#### Protein expression and purification

Full-length human TDG (410 amino acids) and truncated TDG variants were expressed in *Escherichia coli* and purified as described previously (16). Expression plasmids for all truncated TDG variants were generated by deleting the corresponding nucleotides from plasmid pET28a-hTDG (16) using inverse PCR followed by re-ligation of the linearized plasmid. All truncated TDG variants were confirmed to be catalytically active using a 5fC-containing DNA duplex as previously reported (16). The codon optimized gene fragments for expression of LANA-TDG_1–110_, LANA-TDG_309–410_, and full-length human GADD45a (UniProt identifier: P24522) were purchased as gBlock Gene Fragments from IDT and assembled by PCR as recommended by the manufacturer. The assembled DNA was cloned into the pET28a expression vector (Novagen) between the HindIII and NdeI restriction sites, generating plasmids pET28-LANA.1–110^TDG^, pET28-LANA.309–410^TDG^, and pET28a-GADD45, respectively. Correct assembly of all plasmids was verified by DNA sequencing (Eton Bio, San Diego, CA, USA).

LANA-TDG fusion proteins were expressed and purified as other TDG variants, except no activity screening was carried out. For GADD45a preparation, the plasmid assembled above (pET28a-GADD45) was transformed into BL21 (DE3) cells and the outgrowth (0.8 ml) was used to seed 1 × 100 ml cultures of 2YT media suuplemented with 50 μg/ml Kanamycin. After shaking overnight at 37°C, 25 ml of overnight culture was used to innoculate 4 × 1 l of 2YT media supplemented with 50 ug/ml Kanamycin. The cells were grown to an OD_600_ ∼0.800 at 37°C with vigorous shaking and expression was induced with 0.2 mM IPTG at 37°C for 5 h. Next, the cells were pelleted by centrifugating at 3900 RPM for 60 min using a swinging bucket rotor (4°C) and the resulting cell pellet was resuspended in Buffer H10 (50 mM HEPES, pH 8.0, 300 mM NaCl, 10% glycerol, 10 mM imidazole, 5 mM BME); 40 ml of Buffer H10 was used per 1 l of cell culture. To begin lysis, the resuspended cell pellet was frozen at –80°C (until solid) and then thawed on water at 4°C. The cells were further lysed by sonicating on ice for 5 min, and lysates were cleared by centrifugation (10 000 × g, 60 min) and subsequently filtered with a 0.2 um syringe tip filter. The filtered lysate was applied to a 5 ml HisTrap FF column (GE Healthcare Lifesciences) equilibrated with 5 column volumes (CV) of Buffer H10. The protein-bound resin was then washed with a 10 CV Buffer H10, then GADD45 was eluted with a linear gradient (0 →100%) of buffer H1000 (buffer H10 supplemented with 1 M imidazole) over 10 CV. Fractions containing pure protein were combined and exchanged into Buffer HP50 (50 mM HEPES, pH 8, 50 mM NaCl 10% glycerol, 10 mM BME, 1 mM PMSF) using a 5 ml HiTrap Desalting column (GE Healthcare Lifesciences). Protein samples were stored at –80°C until use.

#### Preparation of DNA templates

The DNA templates (**12-601** and **12-601-FRET**) used to reconstitute nucleosome arrays consisted of 12 copies of the ‘Widom 601′ positioning sequence, each of which is separated by 30 bp of linker DNA (Supplementary Figure S1). These DNAs were assembled as previously described (16,20). See Supplementary Figure S1 caption for details.

#### Reconstitution of mononucleosomes and nucleosome arrays

Reconstitution of both mononucleosomes and nucleosome arrays was carried out via slow salt dialysis as before (20) using histone octamers described above. Immediately following the reconstitution step, samples were centrifuged at 13 000 × RPM for 20 min and the resulting pellets were discarded. Soluble chromatin substrates were stored at 4°C in buffer NB (25 mM NaCl, 0.1 mM PMSF, 10 mM HEPES, pH 7.8) for later use. Reconstituted arrays were analyzed by 0.6% agarose gel electrophoresis (Supplementary Figures S1b, S1e, S5c and S6b) and reconstituted mononucleosomes were analyzed by 5% native PAGE (59:1 acrylamide:bisacrylamide) (Supplementary Figure S2) to check for free DNA. Nucleosome arrays reconstituted from DNA template **12-601** and **12–601-FRET** (Supplementary Figure S1) are referred to as **12-NCP** and **12-NCP-FRET**, respectively.

In the case of human genomic DNA, reconstitution by salt dialysis was conducted as described above, however, the histone octamer:DNA ratios were varied more broadly (0.5:1 – 3.0:1) to identify a suitable ratio for producing soluble nucleosome arrays. In our hands, a ratio of 0.75:1 (octamer:DNA) most efficiently reconstituted fragmented human genomic DNA into chromatin (Supplementary Figure S5).

#### Nucleosome occupancy assay

Nucleosome saturation of arrays was confirmed by digestion of ∼150 ng (∼ 120 fmol) of reconstituted arrays with 7.5 units PflMI and BstXI in buffer NB supplemented with 2 mM MgCl_2_. Free 12-601 DNAs were also digested under the same conditions, and both sets of samples (naked DNA and arrays) were analyzed side-by-side with native PAGE (5%, 59:1, acrylamide: bisacrylamide) (Supplementary Figure S1c, S1f and S5c). Prior to gel loading, the final glycerol concentration was adjusted to 5% using a solution consisting of nucleosome buffer supplemented with 30% glycerol. The presence of a nucleosome band as well as the absence of significant free DNA (<1%) demonstrates full nucleosome occupancies in these reconstituted arrays.

#### Micrococcal nuclease digestion of free and bound arrays

The presence of well positioned 147 bp-nucleosomes was confirmed for **12-NCP** arrays by complete micrococcal nuclease (MNase) digestion (Supplementary Figure S5c). Arrays (150 ng) were digested with 12 units of MNase, in a 20 μl reaction, for 10 min at 37°C in buffer NB supplemented with 0.1 mM MgCl_2_. Reactions were stopped with the addition of SDS loading buffer (LB) to final concentrations of 0.1% SDS and 5% glycerol. The fully digested DNA was analyzed on a 1% agarose gel and visualized by ethidium bromide staining. Nucleosome occupancy following reconstitution of human genomic DNA was confirmed in a similar manner (Supplementary Figure S5c).

**Figure 1.**
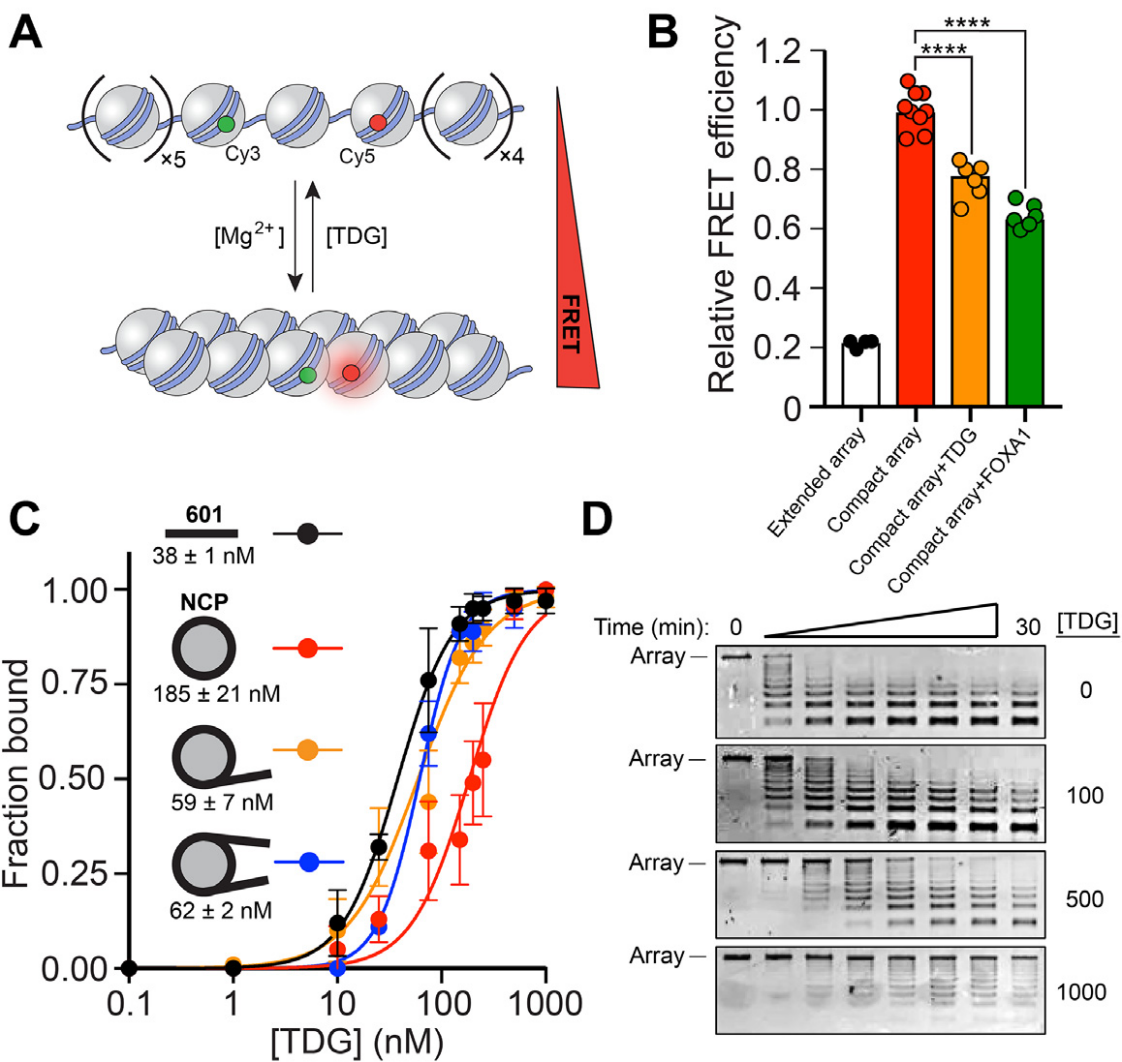
TDG binds and locally opens chromatin fibers. (**A**) Intra-array FRET-based assay to measure the extent of chromatin fiber compaction (16). (**B**) FRET analysis of compact 12-mer arrays (2 mM Mg^2+^) in the presence of TDG (200 nM) or FOXA1 (1 μM). FRET efficiency was normalized to the compact array sample. The extended array sample does not contain Mg^2+^ in the buffer. Raw FRET efficiency is provided in Supplementary Figure S1h. ****P < 0.0001. (**C**) Saturation plots for binding of TDG to naked 601 DNA or mononucleosomes having different arrangements of linker DNA. The *K*_d_ is listed below each substrate. Error bars represent standard deviation from at least three independent experiments. (**D**) MNase digestion of nucleosome arrays in the presence of TDG. The concentration of TDG (nM) used in each experiment is listed to the right.

For the TDG protection assay (Figure 1D), 12-mer arrays (5 nM) were pre-incubated with 0.1, 0.5, or 1 μM TDG in a 25 μl reaction mixture containing buffer NB supplemented with 0.2 mM MgCl_2_ for 15 min at 37°C. At that point, 2.7 μl of MNase reaction buffer (1.5 U/μl MNase and 0.2 mM MgCl_2_) was added and the reaction was allowed to proceed at 37°C. Aliquots were taken at the indicated times and quenched with SDS LB as before. Digestion reactions were analyzed by 0.7% agarose (1 × sodium borate (SB) buffer, 195 V, 25 min) and visualized by ethidium bromide staining.

#### Analysis of chromatin fiber compaction via intra-fiber FRET

Intra-fiber compaction experiments were carried out as previously reported using an identically labeled nucleosome array (**12-NCP-FRET**) (Supplementary Figure S1) (16). Briefly, 10 nM **12-NCP-FRET** arrays were equilibrated in buffer NB supplemented with 2 mM MgCl_2_ at 37°C for 5 min. At that point, either 200 nM TDG or 500 nM FOXA1 was added, and the reaction mixture was incubated for an additional 20 min at 37°C prior to being transferred to a Nunc 384-Well Optical (glass) Bottom Plate (Thermofisher). The plate was imaged using a Typhoon multimode imager (GE Healthcare Lifesciences), and fluorescence intensities were corrected for spectral overlap and direct acceptor excitation as previously described (16,23,24). FRET efficiency (*E*) was calculated via the equation below:

$$\begin{equation*}E\; = \;\frac{{{{{F}}_{{\rm{corr}}}}}}{{{{{F}}_{{\rm{corr}}}} + {{\gamma D}}}}\end{equation*}$$

where *F*_corr_ and *D* are corrected intensities from the transfer and donor channels, respectively, and *γ* represents the detection factor as described in the Supplementary Methods section.

#### Electrophoretic mobility shift assays (EMSA)

The binding affinity of TDG to naked DNA and mononucleosomes was determined by EMSA (Supplementary Figure S2). 5′-[^32^P]-labeled 601 DNA or mononucleosomes (5 nM) were incubated with the indicated concentrations of TDG (0–1 μM) in buffer NB supplemented with 0.2 mM MgCl_2_ and 5% glycerol. The binding reactions were carried out at 37°C for 20 min and were resolved by 5% native PAGE (59:1 acrylamide:bisacrylamide), which were run at 160 V for 30 min at 4°C. The gel was visualized using a GE Typhoon gel imager and quantified using ImageQuant TL software imager (GE Healthcare Lifesciences).

#### Analysis of chromatin oligomerization via precipitation

Chromatin oligomerization was determined by precipitation as previously described (22,25). Briefly, nucleosome arrays (5 nM) were incubated in the presence of the indicated protein in a reaction mixture consisting of buffer NB for 10 min at 37°C. Unless indicated otherwise (Supplementary Figure S3a), Mg^2+^ (MgCl_2_) was not included in the reaction mixture. Following the incubation, samples were centrifuged (13 000 RPM) at 4°C for 15 min and an aliquot of the supernatant was combined with SDS LB and analyzed by 0.7% agarose gel electrophoresis (1 × SB Buffer, 195 V, 20 min).

#### Generation of histone tail deleted nucleosome arrays

Nucleosome arrays lacking individual tail domains (Supplementary Figure S4) were reconstituted using **12-601** DNA and recombinant histone octamers, refolded from either H3 or H4 tail-deleted proteins (globular domains only, H3 residues: 38–135 and H4 residues 17–99). Nucleosome arrays lacking all histone tail domains were generated via Trypsin digestion of intact **12-NCP** arrays. Briefly, lyophilized Trypsin was dissolved in 50 mM acetic acid (100 ng/μl) and diluted 1:1 with 250 mM TRSI (pH 7.9). Nucleosome arrays (25 nM) were then digested with 0.5 ng/μl Trypsin at room temperature for 30 minutes. Reactions were quenched by addition of TQ buffer (final concentration: 50 ng/μl aprotinin, 0.25 mg/ml BSA, 10 mM HEPES pH 7.8, 25 mM NaCl, 0.1 mM PMSF) and samples were stored on ice until time of use. Successful tail removal was confirmed by native and denaturing gel electrophoresis (Supplementary Figure S4a).

#### Fragmentation of human genomic DNA

Human genomic DNA was thawed on ice, and then 20 μg was diluted into 180 μl of 1.1 × NEB NEXT^®^ dsDNA Fragmentase^®^ buffer. Next, the Fragmentase stock solution was vortexed for 3 s and then 20 μl of enzyme was added directly to the DNA mixture, bringing the total volume to 200 μl. The final reaction mixture was vortexed for an additional 3 s, then incubated at 37°C for 8 min, with gentle vortexing every 2 min. The reaction was quenched by addition of SDS to a final concentration of 0.1%, and successful generation of 0.5–3 kb DNA fragments was confirmed by agarose gel (0.7%) electrophoresis (Supplementary Figure S5a). The DNA was then desalted with an EconoSpin mini spin column (cat. no. 1920-050/250, Epoch Life Sciences) and eluted in Milli-Q H_2_O.

#### Analysis of chromatin oligomerization via inter-fiber FRET

Inter-fiber FRET measurements were acquired as described above for intra-fiber FRET. Details regarding this assay are described in the Supplementary Methods section.

#### Chromatin aggregation reversibility assay

Pre-formed chromatin oligomers were generated by incubating 1 μM TDG (or the indicated TDG variant) with 5 nM 12-mer arrays in a reaction mixture (8 ul) containing buffer NB at 37°C for 15 min. At this point, 4 ul of a solution containing either 601 DNA or GADD45a, both at 3-times the final desired concentration (Supplementary Figures S10 and 11), was added to the mixture. After incubating for 10 min at 37°C, samples were centrifuged at (13 000 RPM) at 4°C for 15 min and an aliquot of the supernatant was combined with SDS LB and analyzed by agarose gel (0.7%) electrophoresis (Supplementary Figures S10 and 11).

#### M.SssI methylation of nucleosome arrays

For methylation reactions, **12-601** DNA (60 nM) was incubated with 0.35 U/μl M.SssI in CutSmart buffer supplemented with 0.4 mM SAM at 37°C for 4 h. To confirm CpG sites were fully methylated, a 75 fmol aliquot was digested with 10 units HpaII in a 10 μl reaction containing 1 × CutSmart buffer at 37°C for 45 min. Following the digest, glycerol was added (5%, v:v) and the reactions were analyzed via agarose gel (0.7%) electrophoresis (Supplementary Figure S12a). HpaII-resistant **12-601** DNA was used in subsequent nucleosome reconstitutions and confirmed to form chromatin via native agarose gel electrophoresis (Supplementary Figure S12b).

#### Statistical analysis

**Figure 2.**
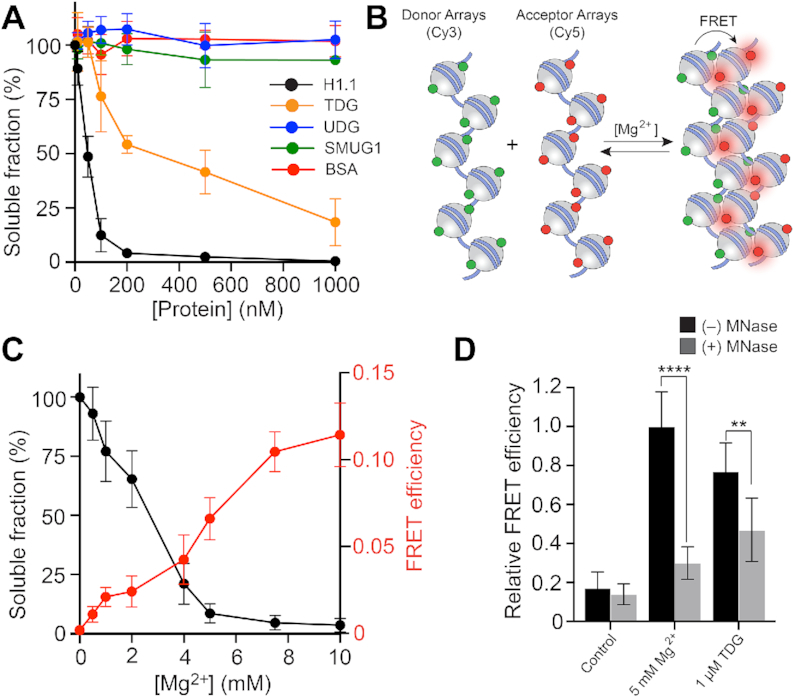
TDG promotes chromatin condensation. (**A**) Precipitation assay to monitor nucleosome array oligomerization. Nucleosome arrays were incubated with the indicated protein, oligomers were removed by centrifugation, and the percentage of arrays remaining in solution was determined by gel electrophoresis. (**B**) FRET-based assay to monitor inter-fiber oligomerization. (**C**) Mg^2+^-induced oligomerization of nucleosome arrays. Precipitation data (black) is shown on the left Y-axis, and inter-fiber FRET efficiency (red) is shown on the right Y-axis. (**D**) Comparison of the inter-fiber FRET efficiency for arrays treated with Mg^2+^ or TDG. Error bars represent standard deviation from at least three independent experiments.

All FRET data were presented as means and standard deviations. Statistical analysis of intra- and inter-fiber FRET studies was conducted using GraphPad Prism (v8.4.2). For comparison of the corrected FRET intensities (Figures 1B and 2D) between samples containing either free **12-NCP** arrays or **12-NCP** arrays that had been oligomerized by TDG or Mg^2+^, all data sets were first compared by unpaired one-way analysis of variance (ANOVA), then significant differences were determined between each condition tested using a Tukey's multiple comparisons test (α = 0.05). An identical analysis (ANOVA and Tukey's multiple comparisons) was used to compare TDG-induced precipitation of wild-type and hyper-methylated arrays (Figure 5).

## RESULTS AND DISCUSSION

### TDG locally opens chromatin structure

We first considered the ability of TDG to alter chromatin structure at the single-fiber level (we use ‘TDG’ throughout to refer to the full-length human protein). In particular, we focused on TDG’s ability to bind to and alter the structure of nucleosome arrays compacted into ‘30 nm’ chromatin fibers. Previous biophysical studies have shown that nonspecific binding of transcription factors (TFs) to nucleosome arrays, and specifically to extra-nucleosomal (or ‘linker’) DNA, causes array decompaction (26). Given TDG’s high affinity for DNA, even in the absence of a target nucleobase (15), we reasoned that TDG may also drive decompaction of 30 nm chromatin fibers through similar interactions. To test this, we assembled 12-mer nucleosome arrays containing fluorescent donor and acceptor dyes that were placed at locations that allow nucleosome stacking interactions, and thus the overall compaction of the array (i.e. formation of 30 nm chromatin fibers), to be monitored by FRET (Förster resonance energy transfer) (Figure 1A and Supplementary Figure S1) (16). Under the conditions used in our assay (2 mM Mg^2+^), nucleosome arrays fold into maximally compact 30 nm fibers (1), which is accompanied by a characteristic increase in FRET efficiency (Figure 1B and Supplementary Figure S1h, i) (16,26). In the presence of 200 nM TDG, FRET efficiency was reduced by ∼20%, indicating that TDG induced decompaction of the arrays. A similar effect was observed for the pioneering TF FOXA1, which is known to actively initiate chromatin decompaction and promote DNA accessibility (27). We presumed that the relevant TDG binding occurred with the linker DNA because, compared to nucleosomal DNA, linker DNA more closely resembles typical B-form DNA (28), is generally more accessible to DNA binding factors (29), and is a better substrate for TDG’s glycosylase activity (16,30). Consistently, we found that TDG binds mononucleosomes containing linker DNA (30 bps) more strongly than those without (*K*_d_ = 59 ± 7 and 185 ± 21 nM, respectively) (Figure 1C), and protects linker DNA within nucleosome arrays from micrococcal nuclease (MNase) digestion (Figure 1D). Overall, these results strongly suggest that TDG drives chromatin fiber decompaction through nonspecific binding to linker DNA.

### TDG promotes chromatin condensation

We next asked whether TDG’s association with chromatin fibers influences their ability to undergo oligomerization (also referred to as ‘condensation’). *In vitro*, individual chromatin fibers undergo self-association into higher-order oligomeric structures at Mg^2+^ concentrations greater than 3–4 mM, a process that mimics the formation of long-range intra- and inter-fiber interactions observed in native chromatin (1,25). We incubated 12-mer nucleosome arrays with increasing concentrations of Mg^2+^, removed the precipitated oligomers by centrifugation and quantified the unassociated fibers in the supernatant. Compared to Mg^2+^ alone, the presence of TDG resulted in a profound increase in array oligomerization (Supplementary Figure S3a). A similar affect was observed for the monovalent cation K^+^ (Supplementary Figure S3b). Notably, significant precipitation of arrays was observed even in the absence of these added salts, suggesting that TDG alone is capable of inducing chromatin condensation (Supplementary Figure S3). Therefore, we excluded Mg^2+^ and K^+^ from the following experiments to ensure that the observed chromatin condensation could be attributed solely to TDG. Indeed, titration of 12-mer arrays with only TDG led to a concentration-dependent increase in precipitated material (Figure 2A and Supplementary Figure S3c), with the midpoint for array oligomerization occurring at ∼200 nM TDG (∼3:1 molar ratio of TDG to mononucleosome). For comparison, the midpoint for histone H1.1-induced oligomerization under the same conditions occurred at ∼50 nM (∼1:1 molar ratio of H1.1 to mononucleosome). The fact that TDG induced both nucleosome array decompaction, as well as inter-fiber oligomerization, at similar concentrations suggests that these two processes are coupled. We chose not to incubate TDG with arrays that had been pre-bound by H1.1 because these proteins are localized to different chromatin domains *in vivo*. For instance, TDG and its substrates, 5fC/5caC, are primarily localized to *active* promoter and enhancers (12,31,32), whereas linker histone H1.1 is depleted at these sites (33). This indicates that TDG will most often be bound to chromatin lacking H1.1. In contrast to TDG, two related DNA glycosylases, uracil DNA glycosylase (UDG) and single-stranded mono-functional uracil glycosylase (SMUG1), as well as BSA, had no effect on chromatin solubility (Figure 2A and Supplementary Figure S3c). TDG-mediated array oligomerization still occurred in the absence of histone N-terminal tail domains (Supplementary Figure S4). Thus, histones tails are not essential for TDG-mediated chromatin condensation. This is in contrast to linker histones, which have been shown to require the histone tail domains to induce oligomerization (34). This suggests that histone H1 and TDG promote chromatin condensation through distinct mechanisms. Although the histone tail domains were mostly dispensable for array oligomerization by TDG, nucleosome cores are essential, as TDG failed to precipitate free 12-mer DNA (Supplementary Figure S4b). Importantly, we found that oligomerization is not coupled to DNA sequence, as TDG precipitated chromatin reconstituted from human genomic DNA (Supplementary Figure S5). Finally, it is worth emphasizing that the nucleosome arrays employed in this study lack substrates for TDG base excision, indicating that catalysis is not a requirement for chromatin condensation.

We further confirmed that the insoluble TDG-complexes were in fact oligomers, comprising multiple chromatin fibers, using an inter-fiber FRET-based assay (Figure 2B). Nucleosome arrays were labelled separately with either Cy3 (donor) or Cy5 (acceptor) dyes via maleimide conjugation to histone H2A bearing a N110C mutation, and the labelled arrays were mixed in a 1:1 ratio. Upon fiber oligomerization, which has been proposed to involve interdigitation of nucleosomes between different fibers (35), the dyes become close enough in space to allow for efficient FRET (22). We first validated the method using Mg^2+^, which is well known to induce chromatin fiber oligomerization. Consistently, titration of the donor/acceptor array mixture with increasing concentrations of Mg^2+^ resulted in a concentration dependent increase in FRET efficiency. Moreover, pre-treatment of the array mixture with MNase resulted in a loss of FRET signal (Figure 2C), which is consistent with the inability of mononucleosomes to undergo Mg^2+^-induced oligomerization (36). These observations strongly suggest that the FRET system properly monitors inter-fiber oligomerization. We then applied the assay to TDG. Treatment of the donor/acceptor array mixture with 1 uM TDG, which induces nearly complete array precipitation (Figure 2A), resulted in a pronounced increase in inter-fiber FRET relative to untreated arrays (Figure 2D). Furthermore, the inter-fiber FRET efficiency of TDG treated arrays was similar to arrays treated with 5 mM Mg^2+^, which also induces complete array precipitation. Collectively, these data indicate that the observed precipitation of nucleosome arrays by TDG is indeed due to inter-fiber oligomerization. Interestingly, incubation of MNase-treated arrays (i.e. mononucleosomes) with TDG resulted in a modest reduction in the inter-molecular FRET compared to what was observed for Mg^2+^ (5 mM) (Figure 2D). This difference suggests that oligomerization by TDG involves more specific bridging interactions between nucleosomes and TDG, and is consistent with our observation that, despite binding tightly to DNA, TDG requires nucleosome cores to induce array oligomerization.

### The N- and C-terminal domains of TDG have opposing roles during chromatin condensation

**Figure 3.**
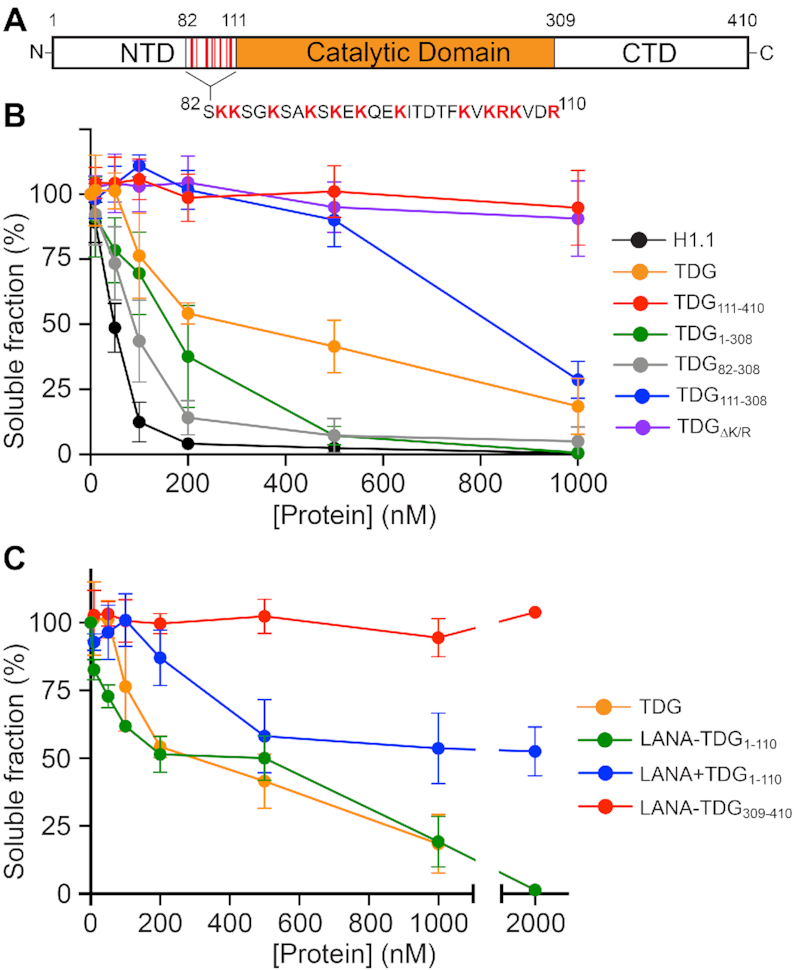
TDG-mediated chromatin oligomerization is dependent on its N- and C-terminal domains. (**A**) TDG domains discussed in this work. (**B**, **C**) Precipitation assay to monitor nucleosome array oligomerization. Nucleosome arrays were incubated with the indicated protein, oligomers were removed by centrifugation, and the percentage of arrays remaining in solution was determined by gel electrophoresis. Error bars represent standard deviation from at least three independent experiments.

The linker histone H1.1 contains a disordered positively charged C-terminal domain (CTD) that is responsible for stabilizing secondary chromatin structures and chromatin condensation (17–19). The N-terminal domain (NTD) of TDG (residues 1–110; Figure 3A), which confers enhanced DNA binding relative to its absence (37) and has other important regulatory functions (9,10), shares a number of similarities with the histone H1.1 CTD: they are both highly basic, mostly disordered, and have low sequence complexity (Supplementary Figure S7). This observation prompted us to ask whether TDG’s NTD is responsible for mediating chromatin condensation. Indeed, we found that deletion of residues 1–110 (TDG_111–410_) completely abolished TDG’s ability to condense nucleosome arrays (Figure 3B and Supplementary Figure S8). Interestingly, deletion of TDG’s CTD (residues 309–410; TDG_1–308_ and TDG_82–308_) had the opposite effect, instead promoting array oligomerization in the presence of the NTD. Notably, TDG_82–308_, which contained only the catalytic domain and a particularly basic region of the NTD (residues 82–110), was capable of condensing chromatin with nearly the same efficiency as histone H1.1. Deletion of all lysine and arginine residues from this basic region (red amino acids in Figure 3A) completely abrogated TDG-mediated array oligomerization (Figure 3B; TDG_ΔK/R_). Given that the catalytic domain alone poorly oligomerized arrays (TDG_111–308_), this suggests that TDG’s ability to condense chromatin is localized to residues 82–110 of the NTD, and specifically, the basic side chains. Previous studies using short DNA duplexes have shown that these residues form high-affinity non-specific complexes with DNA (37,38). Thus, in the context of chromatin, we propose that residues 82–110 bind DNA between arrays through non-specific electrostatic interactions to facilitate oligomerization. Our observation that TDG-mediated oligomerization is impaired by the presence of its full N- and C-terminal domains provides additional support for this mechanism, as those domains have been shown to destabilize non-specific interactions between residues 82–110 and DNA (38). This also suggests an important regulatory role for the CTD, as TDG-mediated chromatin condensation is significantly enhanced in its absence. We cannot rule out that TDG dimerization, which has been observed at very high TDG concentrations (>1 uM) (39), contributes to array oligomerization. If so, our results suggest that the CTD may also destabilize this interaction (compare TDG_111–308_ to TDG_111–410_) (Figure 3B).

In order to gain further support for a mechanism involving TDG’s NTD, we tethered residues 1–110 to a nucleosome-targeting peptide from the Kaposi's sarcoma-associated herpesvirus protein LANA (LANA-TDG_1–110_) (19,40). Remarkably, incubating 12-mer arrays with the LANA-TDG_1–110_ fusion protein induced oligomerization to a similar extent as full-length TDG (Figure 3C and Supplementary Figure S9). This effect was dependent on the attachment of TDG_1–110_ to LANA, as proteolytic cleavage of their linkage significantly impaired array oligomerization (LANA + TDG_1–110_). These results not only confirm that TDG-mediated oligomerization is derived largely from its disordered NTD, but also suggests that TDG’s folded catalytic domain (i.e. the ‘reader’ domain) serves in this context to recruit the lysine-rich NTD to chromatin. These data are also in agreement with the previous finding that the NTD binds DNA regardless of whether or not it is attached to TDG, and through similar interactions (38). As expected, a fusion protein comprising LANA and TDG’s CTD (LANA–TDG_309–410_) had no effect on array solubility.

### TDG-mediated chromatin condensation is reversible

**Figure 4.**
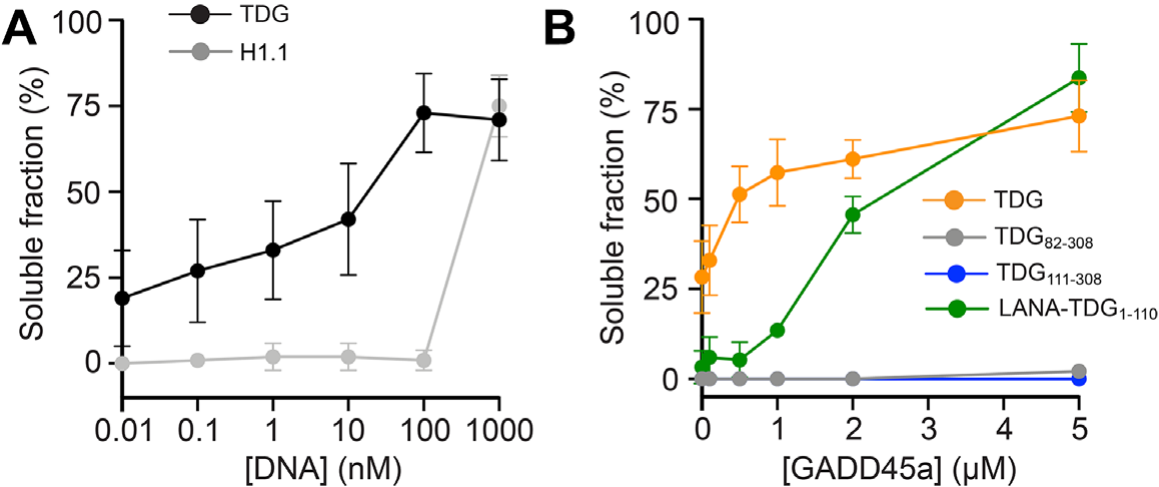
TDG-mediated chromatin oligomerization is reversible. Insoluble chromatin oligomers were incubated with the indicated concentration of 601 DNA (**A**) or GADD45a (**B**), and the change in solubility was monitored following centrifugation. Error bars represent standard deviation from at least three independent experiments.

If chromatin fiber oligomerization is driven by non-specific inter-fiber interactions between TDG’s NTD and DNA, it should be possible to disrupt, and thus re-solubilize, the resulting oligomers using competitor DNA. To test this, we treated insoluble TDG-chromatin oligomers with increasing concentrations of free 207 bp 601 DNA and measured the soluble fraction. Consistent with our hypothesis, excess free DNA was capable of reversing array oligomerization by TDG (Figure 4A and Supplementary Figure S10a). Importantly, the 12-mer arrays remained intact throughout the precipitation and redissolution cycle (Supplementary Figure S10b), as does TDG’s catalytic activity (Supplementary Figure S10c). Therefore, like oligomerization by divalent cations (41), TDG-mediated chromatin condensation is freely reversible. Insoluble H1.1-chromatin oligomers were also reversible by free DNA, but this process was much less gradual than for TDG (4a and S10a). This further highlights the different mechanisms used by these two proteins to condense chromatin. Surprisingly, free DNA was unable to re-solubilize arrays that had been precipitated by TDG_1–308_ and TDG_82–308_, indicating that reversibility is highly dependent on the presence of the CTD (Supplementary Figure S10a). This again is consistent with the CTD acting to destabilize inter-array interactions between the NTD (presumably residues 82–110) and DNA, in this case being required to prevent irreversible oligomerization. Moreover, these data imply that reversal of array oligomerization by DNA does not involve TDG’s catalytic domain.

The N- and C-terminal domains of TDG have been shown to mediate interactions with numerous protein partners (9,10). On the basis of our data above, we predict that these interactions might be capable of altering the formation and/or stability (i.e. reversibility) of TDG-mediated chromatin oligomers. We decided to explore this possibility using growth arrest and DNA damage inducible alpha (GADD45a). In addition to its roles in cell growth control, genomic stability, and DNA repair, GADD45a has been shown to functionally and physically interact with TDG to promote removal of 5fC/5caC from genomic DNA (42,43). Importantly, these interactions involve both the N- and C-terminal domains of TDG (residues 1–132 and 178–397, respectively) (42). As before, we exposed insoluble TDG-chromatin oligomers to increasing concentrations of GADD45a and monitored the change in solubility (Figure 4B and Supplementary Figure S11b). We found that GADD45a readily reversed array oligomerization by full length TDG, with nearly half the precipitated arrays becoming re-solubilized in the presence of a stoichiometric amount of GADD45a relative to TDG. GADD45a does not bind DNA or nucleosomes (44,45), indicating that this effect occurred through TDG. Indeed, GADD45a was also capable of reversing array oligomerization by LANA–TDG_1–110_, although with reduced efficiency, revealing that the functional protein-protein interaction involves at a minimum TDG’s NTD. Importantly, GADD45a was unable to re-solubilize H1.1-chromatin oligomers (Supplementary Figure S11b, c), further supporting a specific interaction between TDG and GADD45a. In agreement with our DNA competition experiments, re-solubilization of TDG-array oligomers by GADD45a was dependent on the presence of TDG’s CTD in the context of the full-length protein (Figure 4B), further supporting a model wherein TDG’s CTD potentiates the disruption of NTD-mediated inter-fiber interactions by external regulators. Collectively, these data demonstrate that TDG-mediated chromatin condensation can be regulated through protein-protein interactions involving its NTD (and presumably its CTD), and importantly, implicate GADD45a in controlling chromatin structural organization through its association with TDG. It is worth noting that multiple lysine residues within the N- and C- terminal domains of TDG undergo posttranslational modification (e.g. acetylation, phosphorylation, and SUMOylation), which has been shown to influence TDG’s interactions with DNA and other proteins (46). By extension of our results above, we anticipate that these modifications will also impact the formation and/or stability of TDG-mediated chromatin oligomers, thereby meriting further investigation.

### DNA methylation impairs chromatin condensation by TDG

**Figure 5.**
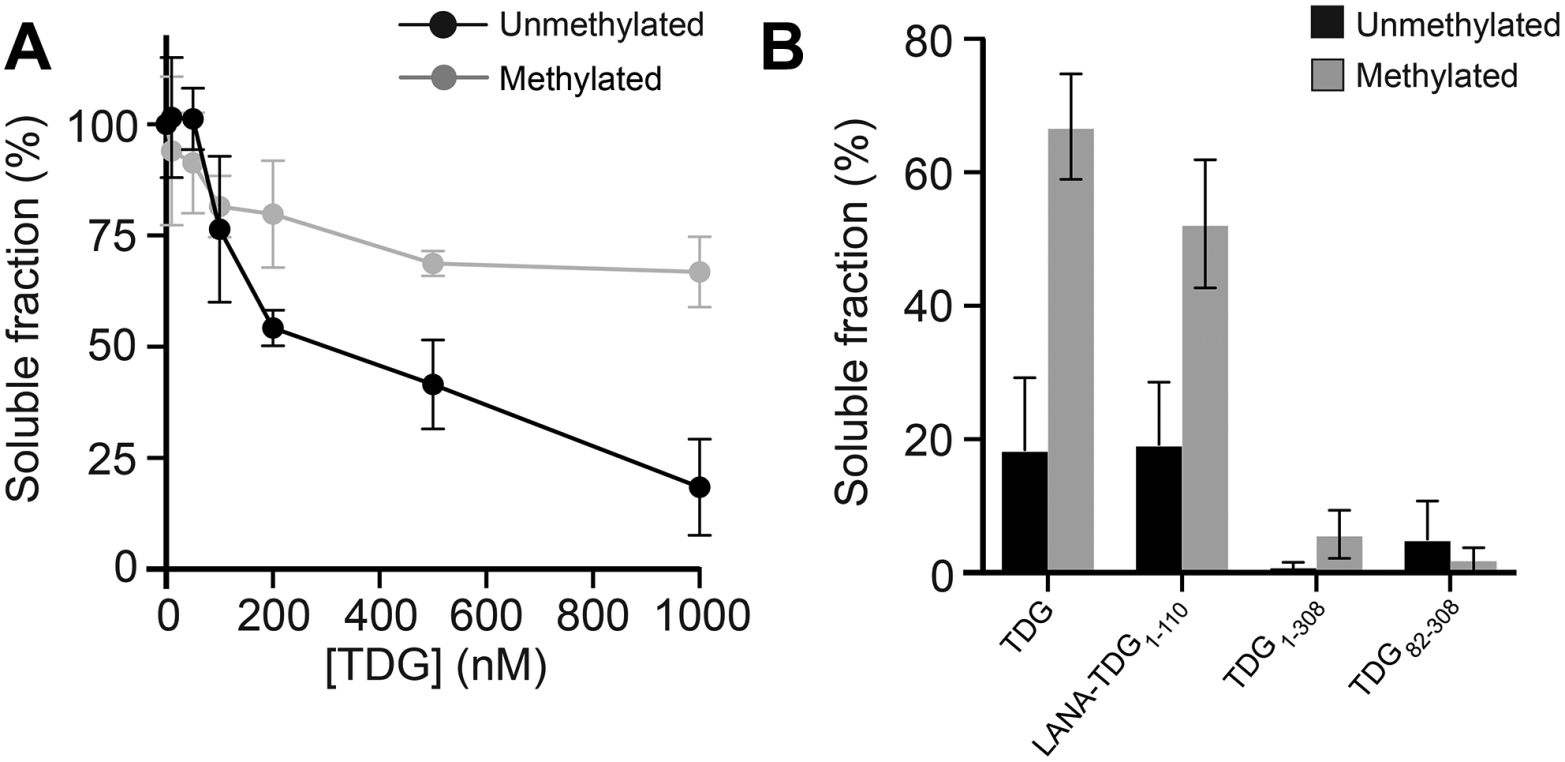
DNA methylation inhibits TDG-mediated chromatin condensation. (**A**) Precipitation assay to monitor nucleosome array oligomerization. (Un)methylated nucleosome arrays were incubated with the indicated concentration of TDG, oligomers were removed by centrifugation, and the percentage of arrays remaining in solution was determined by gel electrophoresis. (**B**) Soluble fraction following treatment of (un)methylated arrays with different TDG variants (1 μM). Error bars represent standard deviation from at least three independent experiments.

Finally, we tested whether TDG-dependent chromatin oligomerization was sensitive to the methylation status of the underlying DNA using nucleosome arrays that had been hypermethylated by the CpG methyltransferase MssSI (Supplementary Figure S12). Whereas DNA methylation only modestly inhibited Mg^2+^-induced array oligomerization (Supplementary Figure S13), methylation drastically impaired array oligomerization by TDG, with the majority of methylated arrays (∼70%) remaining soluble following exposure to 1 μM TDG (Figure 5A). DNA methylation also inhibited chromatin condensation by ^LANA^TDG_1–111_, albeit to a lesser extent than with the full protein. In contrast, TDG variants lacking their CTD were capable of fully aggregating methylated arrays (Figure 5B). Together, these data suggest that DNA methylation weakens inter-array interactions mediated by TDG’s NTD, which is magnified by the destabilizing effects of the CTD. One possible explanation is the increased rigidity imparted on the DNA duplex by 5mC, which has been shown to alter nucleosome stability and dynamics (i.e. DNA accessibility) (47). These changes could, for example, promote intra-array interactions of TDG’s NTD at the expense of inter-array binding and condensation. Additionally, the reduced flexibility of methylated DNA could hinder DNA bending by TDG (48), which may play an important role during condensation (49). We show that DNA binding by TDG is unaffected by methylation (Supplementary Figure S14), ruling out the possibility that TDG’s inability to condense methylated arrays is simply due to impaired binding. Future investigations are required to determine the exact mechanism. It will also be important to examine how other cytosine modifications impact the ability of TDG to condense chromatin, particularly 5fC, which has been shown to greatly enhance DNA flexibility (47). Most excitingly, these data support a potential regulatory mechanism wherein 5mC prevents the formation of TDG-dependent chromatin structures at methylated (or inactive) genomic regions *in vivo*.

## CONCLUSION

**Figure 6.**
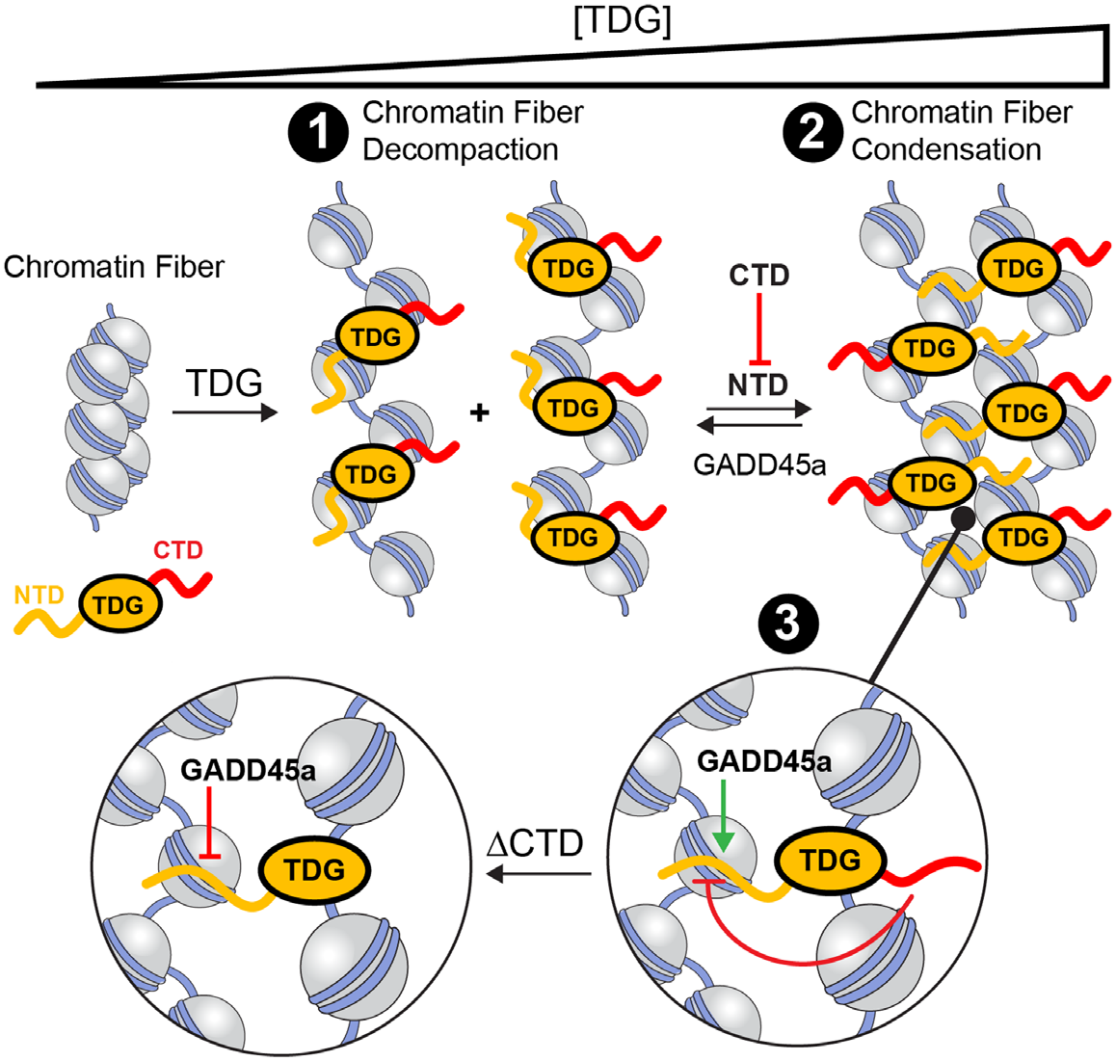
Proposed model for TDG-mediated chromatin remodelling (1). Upon recruitment, TDG preferentially binds to linker DNA between nucleosome resulting in decompaction of the chromatin fiber structure. The basic NTD contributes to nonspecific DNA binding in *cis* (i.e. to the same fiber as the catalytic domain) (37,38). (2) In the presence of nearby chromatin fibers, TDG’s NTD can also bind to DNA in *trans* (i.e. to a different fiber than the catalytic domain), facilitating oligomerization and condensation of the chromatin as local concentration of TDG increase. Because efficient oligomerization requires tethering of the NTD to chromatin (Figure 3C), we propose that DNA binding by the catalytic domain (which requires in *cis* DNA binding by the NTD), along with accompanying array decompaction, precedes oligomerization. (3) The CTD of TDG antagonizes chromatin condensation by weakening inter-fiber interactions between the NTD and DNA, potentially through direct contacts between the two disordered domains (38). This destabilizing affect allows for external regulators (e.g. GADD45a) to bind to and sequester TDG’s NTD away from DNA, resulting in disruption of inter-fiber interactions and re-solubilization of the chromatin. However, in the absence of the CTD’s destabilizing affect (ΔCTD), chromatin condensation becomes non-reversible due to tight inter-fiber binding of the NTD.

In summary, this work provides the first evidence that TDG directly alters chromatin structure through its physical interactions with DNA, thus further expanding TDG’s functional repertoire to include chromatin remodeling. The proposed model depicted in Figure 6 summarizes our finding (see Supplementary Table S1). Given TDG’s involvement in a number of gene regulatory pathways, such as DNA demethylation and transcriptional control, chromatin remodeling by TDG will have important biological consequences. For example, TDG’s intrinsic ability to bind and locally open compact chromatin fibers may play a role in its ability to recruit and/or promote the activity of downstream factors during transcriptional activation, such has been observed for pioneering TFs (50). Importantly, these ‘pioneering’ activities could be targeted to sites enriched with 5fC/5caC, with TDG’s slow off-rate following excision allowing for stable recruitment of activating transcription factors and further chromatin opening (15,51). One particularly exciting possibility offered by our results is that TDG directly participates in the formation of long-range chromatin fiber interactions, for example, between gene enhancers and promoters during transcriptional activation (i.e. chromatin looping). In support of this hypothesis, genome-wide studies have shown that, in response to 17β-estradiol (E2), TDG localizes to sites that are involved in the interactions between promoters and enhancers of E2-responsive genes (12). Importantly, the three-dimensional (3D) reorganization of E2-responsive genes upon E2 stimulation is abrogated upon TDG depletion, indicating that TDG plays a central role in 3D chromosomal rearrangements during E2-mediated transcriptional activation *in vivo*. Importantly, a very recent study showed that insoluble chromatin aggregates formed by salts (e.g. Mg^2+^) and histone H1 *in vitro* are actually liquid-liquid phase-separated droplets (19). Although additional studies are needed to determine the exact nature of the chromatin aggregates formed by TDG, this strongly suggests that they are also a phase-separated liquid. The fact that chromatin condensation by TDG is dependent on its disordered NTD further supports a phase-separation model, as phase separation of proteins is often driven by weak, multivalent interactions between intrinsically disordered protein domains (52–54). The importance of disorder within TDG’s NTD is further reflected in phylogenic analysis of TDG sequence and structure (Supplementary Figure S15), which reveals that, although sequence conservation is low within the NTD, the overall disorder content remains fairly constant across species. A phase-separation model is also consistent with TDG playing an architectural role during transcriptional activation of E2-responsive genes, as this process has been shown to involve the formation of liquid-like phase-separated compartments (or condensates) by the proteins involved (14,52). More broadly, coupling chromatin condensation and phase separation to TDG provides a mechanism whereby such compartments could be targeted to (or be initiated at) genomic sites enriched with 5fC/5caC and, as our data suggests, directed away from methylated chromatin domains. Such a mechanism would directly link DNA epigenetics and BER to genome organization.

## DATA AVAILABILITY

The data generated during all experiments is available from the author upon reasonable request.

## Supplementary Material

gkab040_Supplemental_File

## References

[1] Hansen J.C. Conformational dynamics of the chromatin fiber in solution: determinants, mechanisms, and functions. Annu. Rev. Biophys. Biomol. Struct. 2002; 31:361–392. 11988475 10.1146/annurev.biophys.31.101101.140858

[2] Eagen K.P. Principles of chromosome architecture revealed by Hi-C. Trends Biochem. Sci. 2018; 43:469–478. 29685368 10.1016/j.tibs.2018.03.006 PMC6028237

[3] Wiebauer K. , Jiricny J. Mismatch-specific thymine DNA glycosylase and DNA polymerase beta mediate the correction of G.T mispairs in nuclear extracts from human cells. Proc. Natl. Acad. Sci. U.S.A. 1990; 87:5842–5845. 2116008 10.1073/pnas.87.15.5842 PMC54424

[4] Neddermann P. , Jiricny J. The purification of a mismatch-specific thymine-DNA glycosylase from HeLa cells. J. Biol. Chem. 1993; 268:21218–21224. 8407958

[5] Maiti A. , Drohat A.C. Thymine DNA glycosylase can rapidly excise 5-formylcytosine and 5-carboxylcytosine: Potential implications for active demethylation of cpg sites. J. Biol. Chem. 2011; 286:35334–35338. 21862836 10.1074/jbc.C111.284620 PMC3195571

[6] He Y.-F. , Li B.-Z., Li Z., Liu P., Wang Y., Tang Q., Ding J., Jia Y., Chen Z., Li L. et al. Tet-mediated formation of 5-carboxylcytosine and its excision by TDG in mammalian DNA. Science. 2011; 333:1303–1307. 21817016 10.1126/science.1210944 PMC3462231

[7] Drohat A.C. , Coey C.T. Role of base excision “repair” enzymes in erasing epigenetic marks from DNA. Chem. Rev. 2016; 116:12711–12729. 27501078 10.1021/acs.chemrev.6b00191 PMC5299066

[8] Schuermann D. , Weber A.R., Schär P. Active DNA demethylation by DNA repair: Facts and uncertainties. DNA Repair (Amst.). 2016; 44:92–102. 27247237 10.1016/j.dnarep.2016.05.013

[9] Cortázar D. , Kunz C., Saito Y., Steinacher R., Schär P. The enigmatic thymine DNA glycosylase. DNA Repair (Amst.). 2007; 6:489–504. 17116428 10.1016/j.dnarep.2006.10.013

[10] Sjolund A.B. , Senejani A.G., Sweasy J.B. MBD4 and TDG: multifaceted DNA glycosylases with ever expanding biological roles. Mutat. Res. 2013; 743–744:12–25. 10.1016/j.mrfmmm.2012.11.001 PMC3661743 23195996

[11] Tini M. , Benecke A., Um S.-J., Torchia J., Evans R.M., Chambon P. Association of CBP/p300 acetylase and thymine DNA glycosylase links DNA repair and transcription. Mol. Cell. 2002; 9:265–277. 11864601 10.1016/s1097-2765(02)00453-7

[12] Kolendowski B. , Hassan H., Krstic M., Isovic M., Thillainadesan G., Chambers A.F., Tuck A.B., Torchia J. Genome-wide analysis reveals a role for TDG in estrogen receptor-mediated enhancer RNA transcription and 3-dimensional reorganization. Epigenet. Chromatin. 2018; 11:5. 10.1186/s13072-018-0176-2 PMC5787930 29378668

[13] Hassan H.M. , Kolendowski B., Isovic M., Bose K., Dranse H.J., Sampaio A.V., Underhill T.M., Torchia J. Regulation of active DNA demethylation through RAR-mediated recruitment of a TET/TDG complex. Cell Rep. 2017; 19:1685–1697. 28538185 10.1016/j.celrep.2017.05.007

[14] Nair S.J. , Yang L., Meluzzi D., Oh S., Yang F., Friedman M.J., Wang S., Suter T., Alshareedah I., Gamliel A. et al. Phase separation of ligand-activated enhancers licenses cooperative chromosomal enhancer assembly. Nat. Struct. Mol. Biol. 2019; 26:193–203. 30833784 10.1038/s41594-019-0190-5 PMC6709854

[15] Morgan M.T. , Maiti A., Fitzgerald M.E., Drohat A.C. Stoichiometry and affinity for thymine DNA glycosylase binding to specific and nonspecific DNA. Nucleic Acids Res. 2011; 39:2319–2329. 21097883 10.1093/nar/gkq1164 PMC3064789

[16] Deckard C.E. , Banerjee D.R., Sczepanski J.T. Chromatin structure and the pioneering transcription factor FOXA1 regulate TDG-mediated removal of 5-formylcytosine from DNA. J. Am. Chem. Soc. 2019; 141:14110–14114. 31460763 10.1021/jacs.9b07576

[17] Hendzel M.J. , Lever M.A., Crawford E., Th’ng J.P.H. The C-terminal domain is the primary determinant of histone H1 binding to chromatin in vivo. J. Biol. Chem. 2004; 279:20028–20034. 14985337 10.1074/jbc.M400070200

[18] Allan J. , Mitchell T., Harborne N., Bohm L., Crane-Robinson C. Roles of H1 domains in determining higher order chromatin structure and H1 location. J. Mol. Biol. 1986; 187:591–601. 3458926 10.1016/0022-2836(86)90337-2

[19] Gibson B.A. , Doolittle L.K., Schneider M.W.G., Jensen L.E., Gamarra N., Henry L., Gerlich D.W., Redding S., Rosen M.K. Organization of chromatin by intrinsic and regulated phase separation. Cell. 2019; 179:470–484. 31543265 10.1016/j.cell.2019.08.037 PMC6778041

[20] Banerjee D.R. , Deckard C.E., Elinski M.B., Buzbee M.L., Wang W.W., Batteas J.D., Sczepanski J.T. Plug-and-play approach for preparing chromatin containing site-specific DNA modifications: the influence of chromatin structure on base excision repair. J. Am. Chem. Soc. 2018; 140:8260–8267. 29883113 10.1021/jacs.8b04063

[21] Muthurajan U. , Mattiroli F., Bergeron S., Zhou K., Gu Y., Chakravarthy S., Dyer P., Irving T., Luger K. In vitro chromatin assembly: strategies and quality control. Methods Enzymol. 2016; 573:3–41. 27372747 10.1016/bs.mie.2016.01.002 PMC5098222

[22] Fierz B. , Chatterjee C., McGinty R.K., Bar-Dagan M., Raleigh D.P., Muir T.W. Histone H2B ubiquitylation disrupts local and higher-order chromatin compaction. Nat. Chem. Biol. 2011; 7:113–119. 21196936 10.1038/nchembio.501 PMC3078768

[23] Gansen A. , Hieb A.R., Böhm V., Tóth K., Langowski J. Closing the gap between single molecule and bulk FRET Analysis of nucleosomes. PLoS One. 2013; 8:e57018. 23637734 10.1371/journal.pone.0057018 PMC3630217

[24] Hieb A.R. , D’Arcy S., Kramer M.A., White A.E., Luger K. Fluorescence strategies for high-throughput quantification of protein interactions. Nucleic Acids Res. 2012; 40:e33. 22121211 10.1093/nar/gkr1045 PMC3299996

[25] Pepenella S. , Murphy K.J., Hayes J.J. Intra- and inter-nucleosomal interactions of the core histone tail domains in higher-order chromatin structure. Chromosoma. 2014; 123:3–13. 23996014 10.1007/s00412-013-0435-8 PMC3938996

[26] Poirier M.G. , Oh E., Tims H.S., Widom J. Dynamics and function of compact nucleosome arrays. Nat. Struct. Mol. Biol. 2009; 16:938–944. 19701201 10.1038/nsmb.1650 PMC2748796

[27] Cirillo L.A. , Lin F.R., Cuesta I., Friedman D., Jarnik M., Zaret K.S. Opening of compacted chromatin by early developmental transcription factors HNF3 (FoxA) and GATA-4. Mol. Cell. 2002; 9:279–289. 11864602 10.1016/s1097-2765(02)00459-8

[28] Schalch T. , Duda S., Sargent D.F., Timothy R.J. X-Ray Structure of a tetranucleosome and its implications for the chromatin fibre. Nature. 2005; 436:138–141. 16001076 10.1038/nature03686

[29] Poirier M.G. , Bussiek M., Langowski J., Widom J. Spontaneous access to DNA target sites in folded chromatin fibers. J. Mol. Biol. 2008; 379:772–786. 18485363 10.1016/j.jmb.2008.04.025 PMC2481406

[30] Tarantino M.E. , Dow B.J., Drohat A.C., Delaney S. Nucleosomes and the three glycosylases: High, medium, and low levels of excision by the uracil DNA glycosylase superfamily. DNA Repair (Amst.). 2018; 72:56–63. 30268365 10.1016/j.dnarep.2018.09.008 PMC6420825

[31] Neri F. , Incarnato D., Krepelova A., Rapelli S., Anselmi F., Parlato C., Medana C., Dal Bello F., Oliviero S. Single-base resolution analysis of 5-formyl and 5-carboxyl cytosine reveals promoter DNA methylation dynamics. Cell Rep. 2015; 10:674–683. 25660018 10.1016/j.celrep.2015.01.008

[32] Wu H. , Wu X., Shen L., Zhang Y. Single-base resolution analysis of active DNA demethylation using methylase-assisted bisulfite sequencing. Nat. Biotechnol. 2014; 32:1231–1240. 25362244 10.1038/nbt.3073 PMC4269366

[33] Izzo A. , Kamieniarz-Gdula K., Ramírez F., Noureen N., Kind J., Manke T., van Steensel B., Schneider R. The genomic landscape of the somatic linker histone subtypes H1.1 to H1.5 in human cells. Cell Rep. 2013; 3:2142–2154. 23746450 10.1016/j.celrep.2013.05.003

[34] Carruthers L.M. , Hansen J.C. The core histone N termini function independently of linker histones during chromatin condensation. J. Biol. Chem. 2000; 275:37285–37290. 10970897 10.1074/jbc.M006801200

[35] Maeshima K. , Rogge R., Tamura S., Joti Y., Hikima T., Szerlong H., Krause C., Herman J., Seidel E., DeLuca J. et al. Nucleosomal arrays self-assemble into supramolecular globular structures lacking 30-nm fibers. EMBO J. 2016; 35:1115–1132. 27072995 10.15252/embj.201592660 PMC4868957

[36] Blacketer M.J. , Feely S.J., Shogren-Knaak M.A. Nucleosome interactions and stability in an ordered nucleosome array model system. J. Biol. Chem. 2010; 285:34597–34607. 20739276 10.1074/jbc.M110.140061 PMC2966075

[37] Coey C.T. , Malik S.S., Pidugu L.S., Varney K.M., Pozharski E., Drohat A.C. Structural basis of damage recognition by thymine DNA glycosylase: Key roles for N-terminal residues. Nucleic Acids Res. 2016; 44:10248–10258. 27580719 10.1093/nar/gkw768 PMC5137436

[38] Smet-Nocca C. , Wieruszeski J.-M., Chaar V., Leroy A., Benecke A. The thymine−DNA glycosylase regulatory domain: Residual structure and DNA binding. Biochemistry. 2008; 47:6519–6530. 18512959 10.1021/bi7022283

[39] Maiti A. , Morgan M.T., Pozharski E., Drohat A.C. Crystal structure of human thymine DNA glycosylase bound to DNA elucidates sequence-specific mismatch recognition. Proc. Natl. Acad. Sci. U.S.A. 2008; 105:8890–8895. 18587051 10.1073/pnas.0711061105 PMC2449336

[40] Barbera A.J. , Chodaparambil J.V., Kelley-Clarke B., Joukov V., Walter J.C., Luger K., Kaye K.M. The nucleosomal surface as a docking station for kaposi's sarcoma herpesvirus LANA. Science. 2006; 311:856–861. 16469929 10.1126/science.1120541

[41] Schwarz P.M. , Felthauser A., Fletcher T.M., Hansen J.C. Reversible oligonucleosome self-association: dependence on divalent cations and core histone tail domains. Biochemistry. 1996; 35:4009–4015. 8672434 10.1021/bi9525684

[42] Li Z. , Gu T.-P., Weber A.R., Shen J.-Z., Li B.-Z., Xie Z.-G., Yin R., Guo F., Liu X., Tang F. et al. Gadd45a promotes DNA demethylation through TDG. Nucleic. Acids. Res. 2015; 43:3986–3997. 25845601 10.1093/nar/gkv283 PMC4417182

[43] Niehrs C. , Schäfer A. Active DNA demethylation by Gadd45 and DNA repair. Trends Cell Biol. 2012; 22:220–227. 22341196 10.1016/j.tcb.2012.01.002

[44] Carrier F. , Georgel P.T., Pourquier P., Blake M., Kontny H.U., Antinore M.J., Gariboldi M., Myers T.G., Weinstein J.N., Pommier Y. et al. Gadd45, a p53-responsive stress protein, modifies DNA accessibility on damaged chromatin. Mol. Cell. Biol. 1999; 19:1673–1685. 10022855 10.1128/mcb.19.3.1673 PMC83961

[45] Sytnikova Y.A. , Kubarenko A.V., Schäfer A., Weber A.N.R., Niehrs C. Gadd45a is an RNA binding protein and is localized in nuclear speckles. PLoS One. 2011; 6:e14500. 21249130 10.1371/journal.pone.0014500 PMC3017548

[46] Carter R.J. , Parsons J.L. Base excision repair, a pathway regulated by posttranslational modifications. Mol. Cell. Biol. 2016; 36:1426–1437. 26976642 10.1128/MCB.00030-16 PMC4859697

[47] Ngo T.T.M. , Yoo J., Dai Q., Zhang Q., He C., Aksimentiev A., Ha T. Effects of cytosine modifications on DNA flexibility and nucleosome mechanical stability. Nat. Commun. 2016; 7:10813. 26905257 10.1038/ncomms10813 PMC4770088

[48] Buechner C.N. , Maiti A., Drohat A.C., Tessmer I. Lesion search and recognition by thymine DNA glycosylase revealed by single molecule imaging. Nucleic Acids Res. 2015; 43:2716–2729. 25712093 10.1093/nar/gkv139 PMC4357730

[49] Shakya A. , King J.T. DNA local-flexibility-dependent assembly of phase-separated liquid droplets. Biophys. J. 2018; 115:1840–1847. 30342746 10.1016/j.bpj.2018.09.022 PMC6303412

[50] Zaret K.S. , Carroll J.S. Pioneer transcription factors: establishing competence for gene expression. Genes Dev. 2011; 25:2227–2241. 22056668 10.1101/gad.176826.111 PMC3219227

[51] Waters T.R. , Gallinari P., Jiricny J., Swann P.F. Human thymine DNA glycosylase binds to apurinic sites in DNA but is displaced by human apurinic endonuclease 1. J. Biol. Chem. 1999; 274:67–74. 9867812 10.1074/jbc.274.1.67

[52] Hnisz D. , Shrinivas K., Young R.A., Chakraborty A.K., Sharp P.A. A phase separation model for transcriptional control. Cell. 2017; 169:13–23. 28340338 10.1016/j.cell.2017.02.007 PMC5432200

[53] Boija A. , Klein I.A., Sabari B.R., Dall’Agnese A., Coffey E.L., Zamudio A.V., Li C.H., Shrinivas K., Manteiga J.C., Hannett N.M. et al. Transcription factors activate genes through the phase-separation capacity of their activation domains. Cell. 2018; 175:1842–1855. 30449618 10.1016/j.cell.2018.10.042 PMC6295254

[54] Boeynaems S. , Alberti S., Fawzi N.L., Mittag T., Polymenidou M., Rousseau F., Schymkowitz J., Shorter J., Wolozin B., Van Den Bosch L. et al. Protein phase separation: A new phase in cell biology. Trends Cell Biol. 2018; 28:420–435. 29602697 10.1016/j.tcb.2018.02.004 PMC6034118

