# Supplementary material for: Reversible chromatin condensation by the DNA repair and demethylation factor thymine DNA glycosylase
Source: Nucleic Acids Res. 2021 Feb 8;49(5):2450–9. doi: 10.1093/nar/gkab040 (PMC7969020; doi:10.1093/nar/gkab040)
Supplement: gkab040_Supplemental_File [file gkab040_supplemental_file.pdf]

Supplementary Information

Reversible Chromatin Condensation by the DNA Repair and Demethylation Factor Thymine DNA Glycosylase

Charles E. Deckard III[1] and Jonathan T. Sczepanski[1,*]

[1]Department of Chemistry, Texas A&M University, College Station, Texas 77843, United States

S1. SUPPLEMENTARY TEXT

Supplementary Methods

Analysis of chromatin fiber compaction via intra-fiber FRET. Intra-fiber FRET experiments were carried out as previously described.[1] Nucleosomes N5 and N7 within the array were fluorescently labeled with Cy3 (donor) and Cy5 (acceptor), respectively, placing the dyes in the identical positions reported by Poirier *et. al.*[2] We refer to the corresponding substrates as **12-601-FRET** and **12-NCP-FRET** for naked DNA and arrays, respectively, throughout the SI text (Figure S1). A detailed description of the DNA sequences for **12-601-FRET**, along with its assembly, has been described in our previous reports.[1,3] To allow for accurate determination of the FRET intensity, three separate reaction mixtures were prepared for each condition tested:

DA: Donor to Acceptor arrays (FRET sample)

DO: Donor only arrays

AO: Acceptor only arrays

Each type of array (10 nM) was equilibrated in buffer NB supplemented with 2 mM $MgCl_2$ at 37 °C for 5 minutes. At that point, either 200 nM TDG or 500 nM FOXA1 was added, and the reaction mixture was incubated for an additional 20 minutes at 37 °C prior to being transferred to a Nunc 384-Well Optical (glass) Bottom Plate (Thermofisher) for imaging. Prior to imaging, optical plates were siliconized with Sigmacote (Sigma Aldrich) according to the manufacturer's instructions. All reaction mixtures were imaged using a Typhoon FLA 9500 multimode imager (GE Healthcare Lifesciences) at 100 μm resolution using the three laser settings and emission filters in the order indicated below:[4]

F: FRET channel (532 nm ex.; 665 nm em.; PMT voltage: 675)
A: Acceptor channel (635 nm ex.; 665 nm em.; PMT voltage: 675)
D: Donor channel (532 nm ex.; 575 nm em.; PMT voltage: 625)

All FRET values reported herein were corrected for spectral overlap as previously described.[1,4] Briefly, FRET (F) intensities were determined using **Eq. 1**, which corrects the raw FRET data (F) for background resulting from donor bleed-through into the acceptor emission (X_d, **Eq. 2**), as well as background from direct acceptor excitation at the donor's excitation wavelength (X_a, **Eq. 3**)

$$(1)\ F_{corr} = F - (D \bullet X_d) - (A \bullet X_a)$$

X_a is obtained from the ratio of F and A intensities with the **AO** sample (**Eq. 2**) and X_d is obtained from the ratio of F and D intensities with the **DO** sample (**Eq. 2**).

$$(2)\ X_a = \frac{F}{A} \qquad (3)\ X_d = \frac{F}{D}$$

Values of Xd and Xa were determined separately for each experiment by measuring the **DO** and **AO** samples under identical reaction conditions as the FRET sample (**DA**).

Absolute FRET efficiency (E) was calculated as previously reported using **Eq. 4**:[4,5]

$$(4)\ E = \frac{F_{corr}}{F_{corr} + \gamma D}$$

where F_{corr} is the corrected FRET intensity, D is the donor intensity, and γ represents the detection factor. Term γ is composed of the detection efficiency of each channel, acceptor labeling efficiency (estimated at 90% based on gel electrophoresis), and the ratio of quantum yields between the dyes. A DNA duplex having the donor (Cy3) and acceptor (Cy5) separated by 16 base pairs served as a FRET standard to estimate the value of γ for our system. The donor-acceptor distance and corresponding FRET efficiency for similar duplexes have been reported previously.[7,8] We measured F_{corr} and D intensities for this FRET standard under identical conditions used above for arrays. The measured intensities of F_{corr} and D, along with the expected FRET efficiency of this duplex, allowed us to solve for the (unknown) detection factor γ.

Analysis of chromatin oligomerization via inter-fiber FRET. In order to monitor FRET between arrays, two separate arrays were reconstituted using histone octamers labeled with either Cy3 (donor arrays) or Cy5 (acceptor arrays) dyes via histone $H2A_{N1110C}$ (Figure S6). Inter-fiber FRET efficiency was then determined as described above for intra-fiber FRET using the following samples for each condition tested:

DA: 1:1 mixture of Donor to Acceptor arrays (FRET sample)

DO: 1:1 mixture of Donor to unlabeled arrays (Donor only)

AO: 1:1 mixture of Acceptor to unlabeled arrays (Acceptor only)

TDG activity assay. TDG's catalytic activity following a cycle of oligomerization was determined by adding 325 fmol of a 5fC-containing 601 DNA duplex (**1-601-5fC49**, described in reference 4) to pre-formed TDG-chromatin aggregates before or after they were incubated at 37 ˚C for 15 or 30 minutes. Abasic sites generated by TDG were then cleaved by the addition of APE1 (35 nM) and 0.1 mM $MgCl_2$ to the reaction mixture. The reactions were incubated at 37 ˚C for 45 minutes, then quenched with loading buffer (5% glycerol, 0.1% SDS, 10 mM HEPES, pH 7.8). Cleaved and intact **1-601-5fC49 DNA** were resolved by 10% (29:1) Native PAGE (Figure S10c).

Analysis of TDG sequence and disorder probabilities. The disorder probability for TDG homologs were computed using Protein Disorder Prediction System (PrDOS)[9] and aligned using the calculated disorder probabilities from the catalytic domain. Mean disorder probability was calculated from the averaged predicted values for the TDG N-terminal domain, which considered every amino acid upstream of the PrDOS minima bordering the catalytic domain from each TDG species; this site corresponds to residue D126 in *h*TDG. Sequence similarities were determined for the same N-terminal residues, and the full-length proteins, with respect to *h*TDG via NCBI Blast protein alignment. Gene IDs are as follows: *S. pombe* (yeast): 2539432, *T. carolina triunguis* (tortoise): 112106684, *X. tropicalis* (tree frog): 448432 *G. fortis* (finch): 102035597, *G. gallus* (chicken): 395510, *C. porcellus* (guinea pig): 100724604, *S. scrofa* (pig):100155184, *P. troglodyte*s (chimpanzee):452188, *H. sapiens* (human) 6996.

S2. SUPPLEMENTARY FIGURES

Figure S1

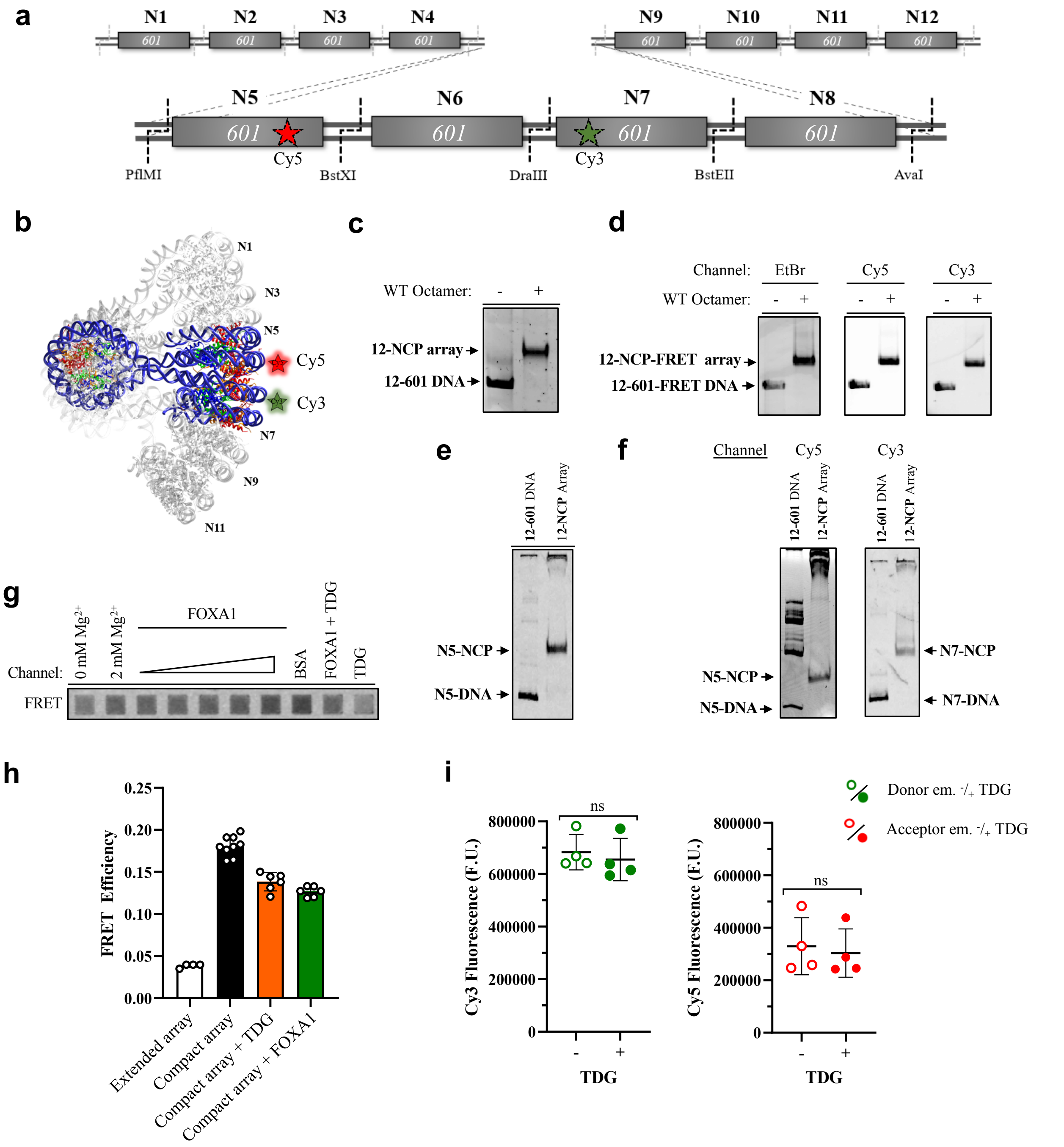

Figure S1. The 12-mer nucleosome arrays used in this work. (a) The DNA template (referred to herein as **12-601**) consists of 12 copies of the “Widom 601” nucleosome positioning sequence, each of which is separated by 30 bp of linker DNA. For intra-fiber FRET experiments, nucleosomes N5 and N7 were fluorescently labeled with Cy3 (donor) and Cy5 (acceptor), respectively, placing the dyes in the identical positions reported by Poirier *et. al*.[2] We refer to this DNA template as **12-601-FRET** throughout the SI. A detailed description of the DNA sequences for **12-601** (previously referred to as **12-601-49**) and **12-601-**

FRET, along with their assembly, has been described in our previous reports.[1,3] Nucleosome arrays reconstituted from DNA template **12-601** and **12-601-FRET** are referred to as **12-NCP** and **12-NCP-FRET**, respectively, throughout the SI. (b) Structural representation of **12-NCP-FRET** arrays used for intra-fiber FRET analysis (pdb: 1zbb, emd: 2600). (c) Native gel analysis (0.6% agarose) of **12-NCP** arrays reconstituted with 1.1 molar equivalent wild-type (WT) histone octamer. (d) The same analysis as in (c) but with **12-NCP-FRET** arrays reconstituted with 1.2 molar equivalent WT octamer. The gel was imaged with the indicated fluorescent channel (EtBr, 532 nm ex.; Filter- LPG. Cy3, 532 nm ex.; Filter- LPG. Cy5, 635 nm ex.; Filter- LPR). (e,f) Restriction digestion analysis (5% native PAGE, 59:1 acrylamide:bisacrylamide) of **12-NCP** (e) and **12-NCP-FRET** (f) arrays demonstrating complete nucleosome occupancy. (g) Representative microplate image of the data depicted in Figure 1b of the main text. Either 0 – 1 μM FOXA1, 1 μM BSA, or 0.2 μM TDG was present. If not otherwise indicated, 2 mM Mg^{2+} was present. (h) Raw FRET efficiencies for the normalized data presented in Figure 1b in the main text. (i) Donor (Cy3) and acceptor (Cy5) emission are unaffected by TDG when present on separate nucleosome arrays. A 1:1 mixture of donor only and acceptor only arrays (10 nM final) were equilibrated in buffer NB supplemented with 2 mM $MgCl_2$ at 37 °C for 5 minutes. At that point, 200 nM TDG was added, and the reaction mixture was incubated for an additional 20 minutes at 37 °C. Cy3 and Cy5 emission was measure as described above.

Figure S2

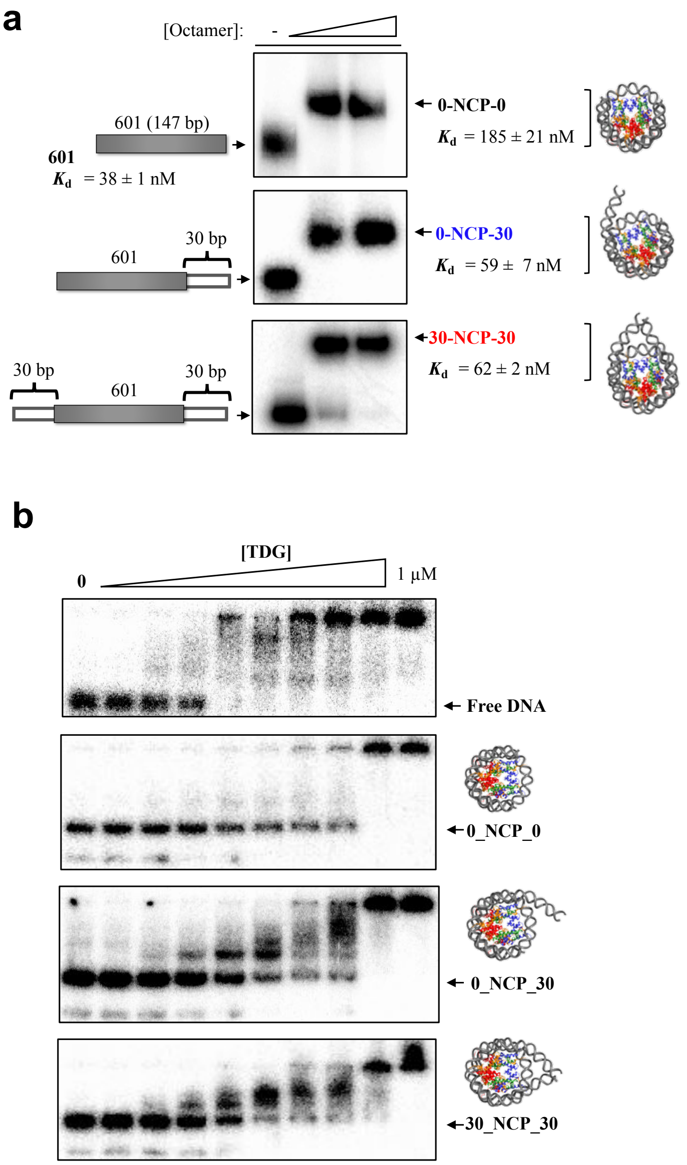

Figure S2. (a) Schematic representation (pdb: 1zbb) and characterization of the mononucleosomes used in this work. Complete reconstitution of all mononucleosome substrates was confirmed by native PAGE (5%, acrylamide:bisacrylamide) analysis. Dissociation constants for TDG binding are provided. (b) Representative gel images of EMSA experiments used to determine the affinity of TDG for different monomucleosomes.

Figure S3

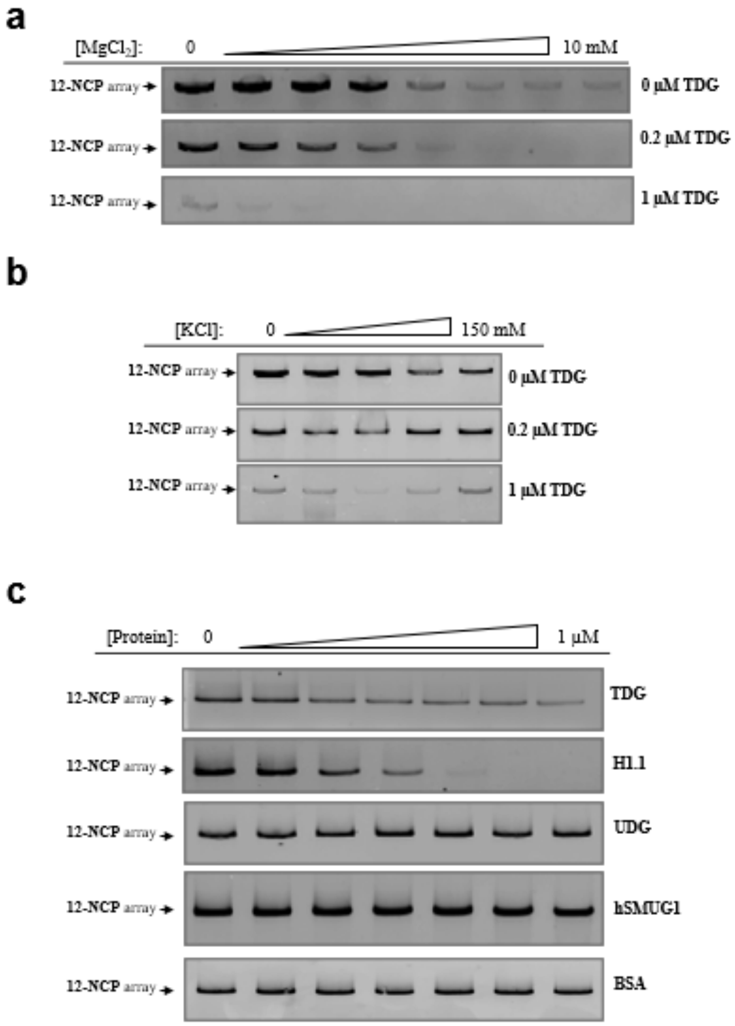

Figure S3. Analysis of chromatin oligomerization via precipitation. (a) Representative gel images showing the soluble fraction from Mg^{2+} dependent (0 – 10 mM) oligomerization experiments carried out in either the presence or absence of TDG. (b) The same experiment depicted in (a), except with 0, 25, 50, 100, or 150 mM KCl. (c) Representative gel images showing the soluble fraction of nucleosome arrays (**12-NCP**) following treatment with various proteins (indicated to the right) in the absence of Mg^{2+} and K^{+}. Reactions were carried out and analyzed as described in the Methods section.

Figure S4

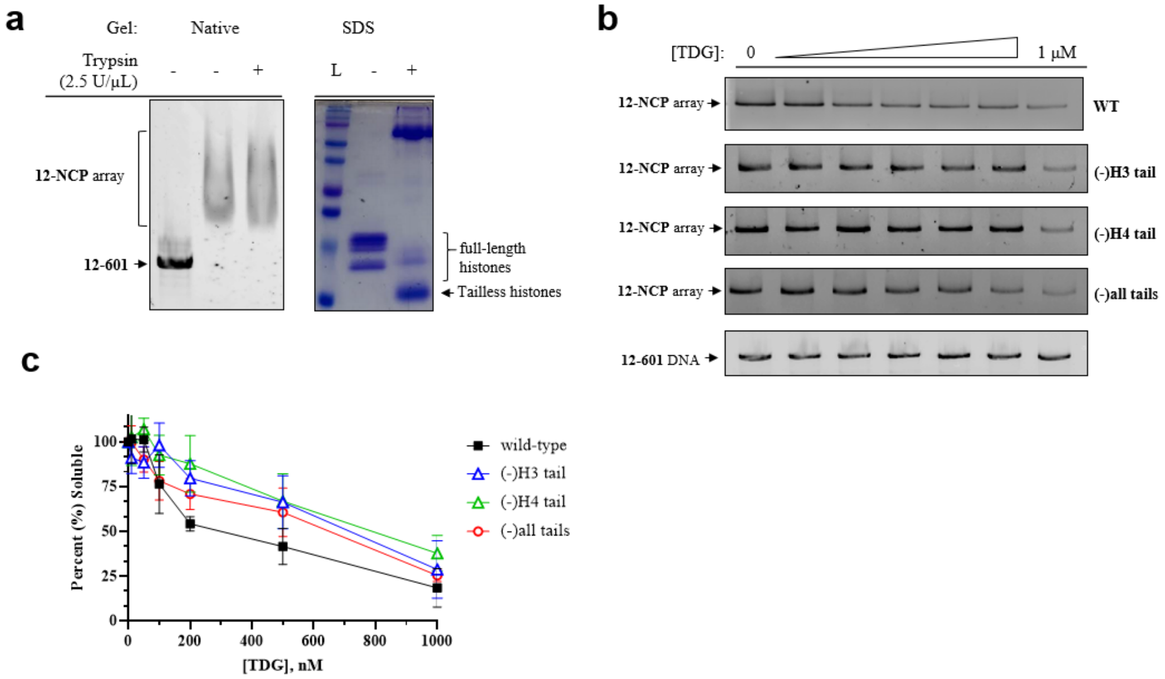

Figure S4. TDG induces chromatin oligomerization in the absence of histone N-terminal tail domains. (a) Agarose gel (0.6%) (left) and SDS PAGE (15%) (right) analysis of **12-NCP** arrays following digestion with Trypsin to remove histone tail domains. (b) Representative gel images showing the soluble fraction of various tailless **12-NCP** arrays (or free **12-601** DNA) following treatment with increasing concentrations of TDG. Histone octamer composition is listed too the right of each gel image. (c) Solubility plot for the experiments shown in part (b). Error bars represent standard deviation from at least three independent experiments.

Figure S5

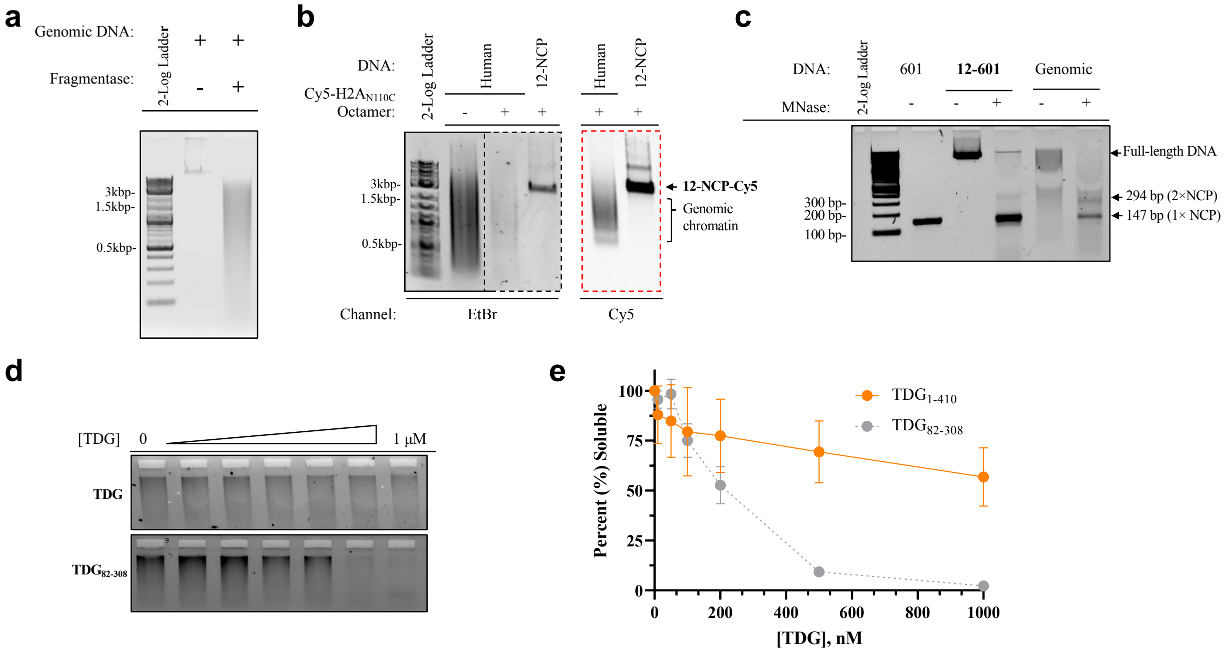

Figure S5. Characterization and analysis of chromatin reconstituted from human genomic DNA. (a) Agarose gel (0.7%) gel analysis of human genomic DNA digested into 0.5-3 kbp fragments with NEB NEXT® dsDNA Fragmentase®, 2-log ladder refers to NEB cat. no. N3200. (b) Agarose gel analysis (0.6%) of chromatin reconstitution reactions using the fragmented genomic DNA depicted in part (a) and histone octamers containing Cy5-labeled $H2A_{N110C}$. A similarly labeled array reconstituted with **12-601** DNA was included for reference (**12-NCP-Cy5**). (c) Agarose gel (1%) analysis confirming the presence of 147 bp nucleosomes in chromatin reconstituted from **12-601** DNA and human genomic DNA. (d) Representative gel images showing the soluble fraction following treatment of chromatin reconstituted from genomic DNA with increasing concentrations of either TDG or $TDG_{82\text{-}308}$. (e) Solubility plot for the experiments depicted in part (d). Error bars represent standard deviation from at least three independent experiments.

Figure S6

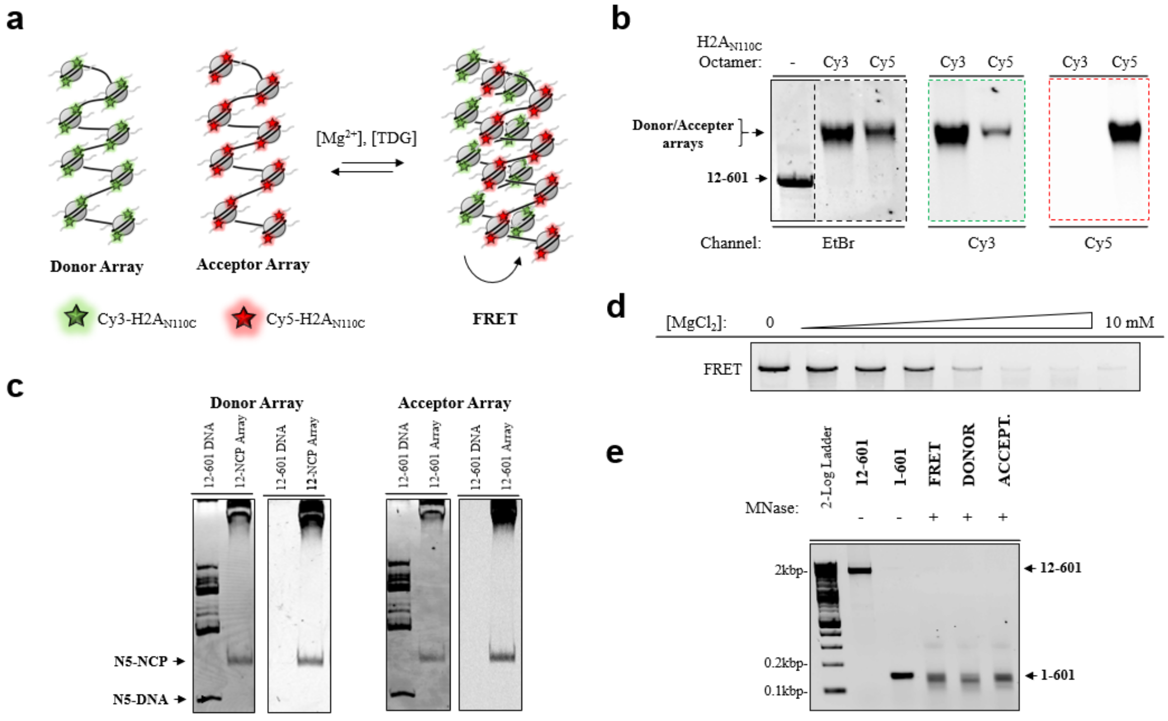

Figure S6. Analysis of chromatin oligomerization via inter-fiber FRET. (a) Schematic depiction of the inter-fiber FRET assay. Two separate nucleosome arrays were labeled with either Cy3 (donor) or Cy5 acceptor) dyes via maleimide conjugation to histone $H2A_{N110C}$. Upon fiber oligomerization, which has been proposed to involve interdigitation of nucleosomes between different fibers[10], the dyes become close enough in space to allow efficient FRET. (b) Agarose gel (0.6%) analysis of donor and acceptor arrays reconstituted from **12-601** DNA template and histone octamers containing either Cy3- or Cy5-labeled histone $H2A_{N110C}$. The gel was visualized using the indicated fluorescent channel. (c) Restriction digestion analysis (5% native PAGE, 59:1 acrylamide:bisacrylamide) of donor and acceptor arrays demonstrating complete nucleosome occupancy. (d) Representative gel image showing the soluble fraction from a Mg^{2+} dependent (0 – 10 mM) oligomerization FRET experiment corresponding to the FRET analysis depicted in Figure 2c. (e) Agarose gel (1%) analysis confirming the digestion of **FRET-**, **DONOR-**, and **ACCEPTOR-** 12mer arrays to individual nucleosome particles via MNase. A fully intact **12-601** and **1-601 DNA** were included for reference.

Figure S7

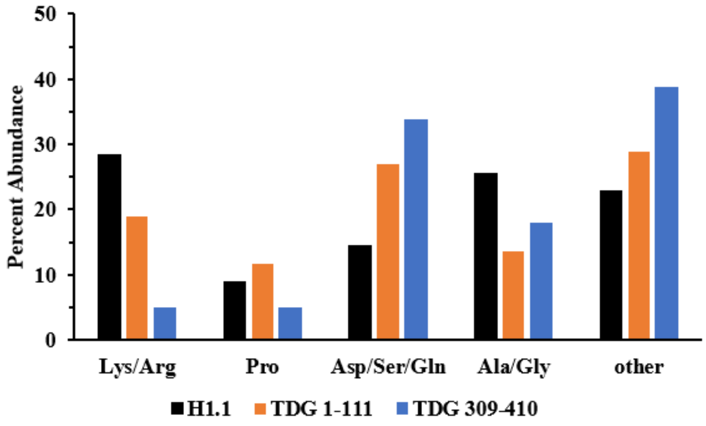

Figure S7. Percent abundance of low complexity amino acids for Histone H1.1 and the indicated TDG tail domains.

Figure S8

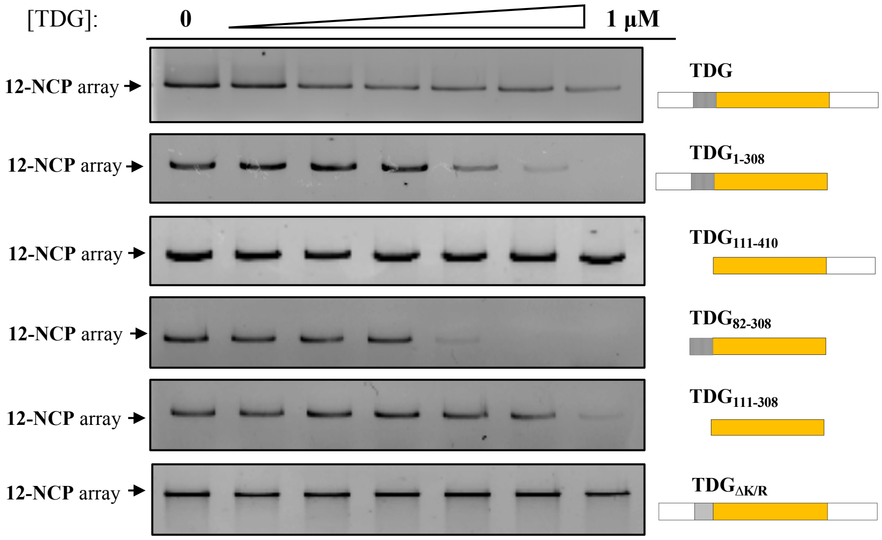

Figure S8. Representative gel images showing the soluble fraction of **12-NCP** arrays following treatment with various TDG variants.

Figure S9

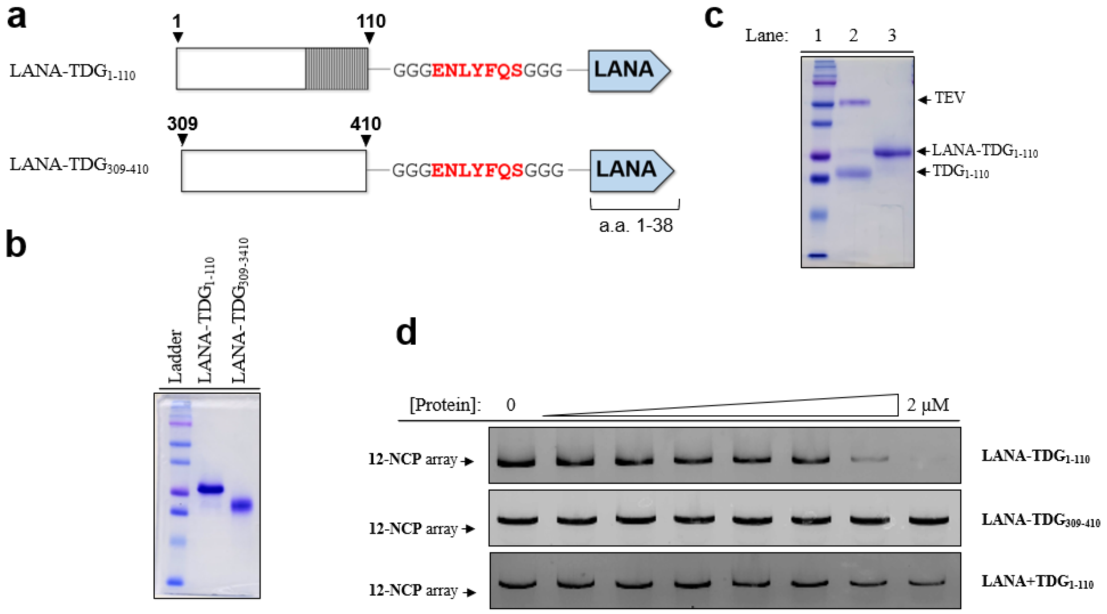

Figure S9. Chromatin oligomerization by TDG-LANA fusion proteins. (a) Schematic depiction of the LANA-TDG fusion proteins used in this work. LANA represents amino acids 1-38 of the latency-associated nuclear antigen 1 protein. (b) Denaturing SDS PAGE (15%, 29:1 acrylamide:bisacrylamide) analysis of purified LANA-TDG proteins stained with Coomassie Brilliant Blue. "Ladder" is the Bio-Rad Precision Plus Pre-stained ladder (cat. no. 1610374). (c) Denaturing SDS PAGE (15%, 29:1 acrylamide:bisacrylamide) analysis of LANA-TDG_{1-110} following treatment with TEV protease to separate the NTD residues 1-110 from the LANA peptide. Lane 1: Bio-Rad Precision Plus Pre-stained ladder; Lane 2: TEV digested LANA-TDG_{1-110} reaction, Lane 3: LANA-TDG_{1-110}. (d) Representative gel images showing the soluble fraction of **12-NCP** arrays following treatment with LANA-TDG proteins depicted in (a).

Figure S10

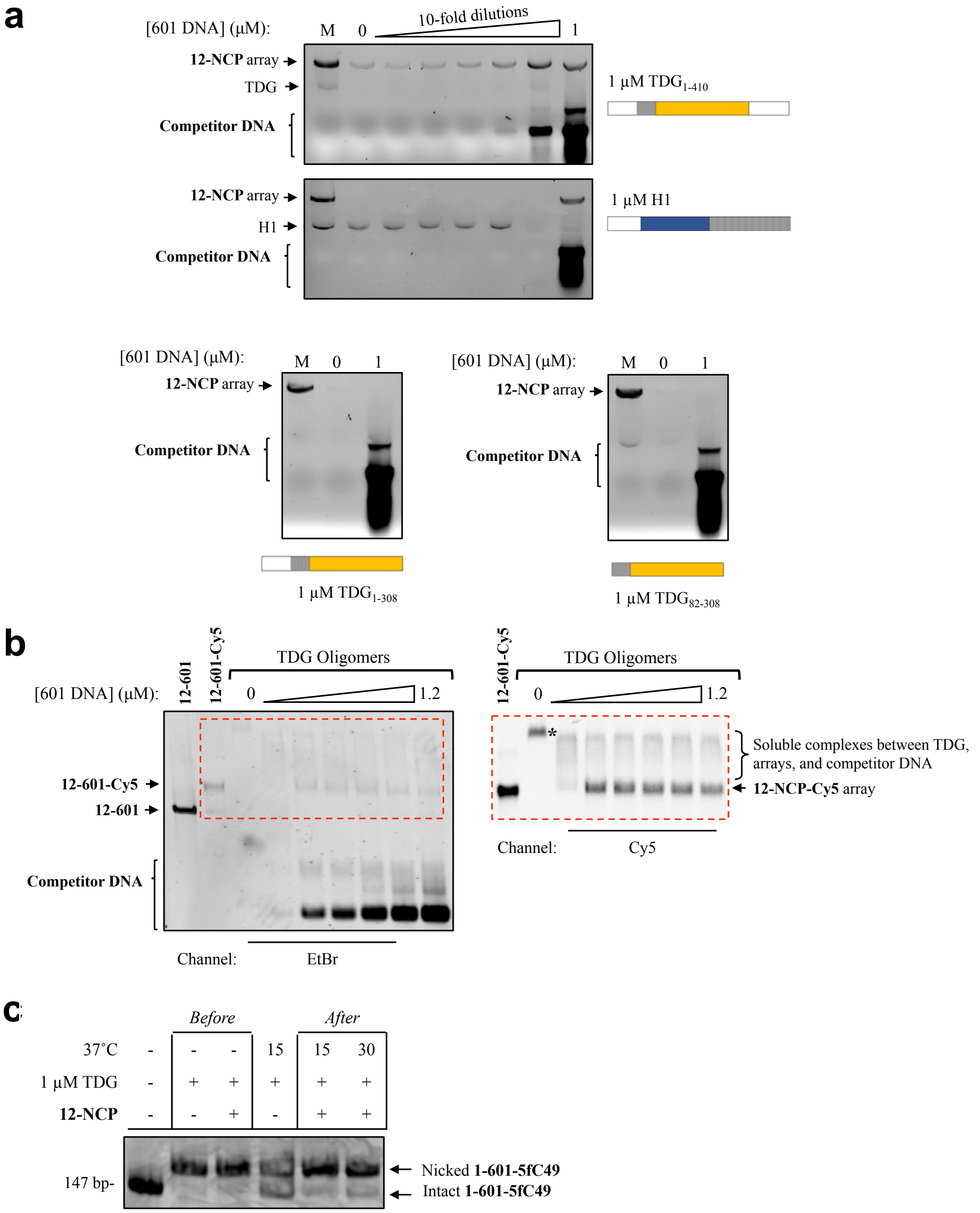

Figure S10. Reversal of insoluble TDG-chromatin oligomers by DNA. (a) Agarose gel analysis of DNA reversibility experiments in which **12-NCP** aggregates, generated with the indicated protein, were exposed to 0-1 μM DNA prior to precipitation. (b) Representative gel images showing the soluble fraction from experiments in which **12-NCP-Cy5** arrays (see Figure S6b) were first aggregated by full-length TDG and subsequently exposed to increased concentrations of 207 bp 601 DNA duplex. Gels were imaged either by EtBr staining (left) or Cy5 fluorescence (right). Asterisk indicates the fractions (~20%) of TDG-bound arrays that remain soluble following the addition of 1 μM full-length TDG (see Figure S8). (c) TDG remains catalytically active following chromatin condensation. TDG activity was determined by adding a 5fC-containing 601 DNA (**1-601-5fC49**) to TDG-chromatin aggregates *before* or *after* they were incubated at 37 ˚C for either 15 or 30 minutes. To facilitate cleavage of abasic sites generated by TDG, APE1 (35 nM) and 0.1 mM $MgCl_2$ was included in the final reaction mixture.

Figure S11

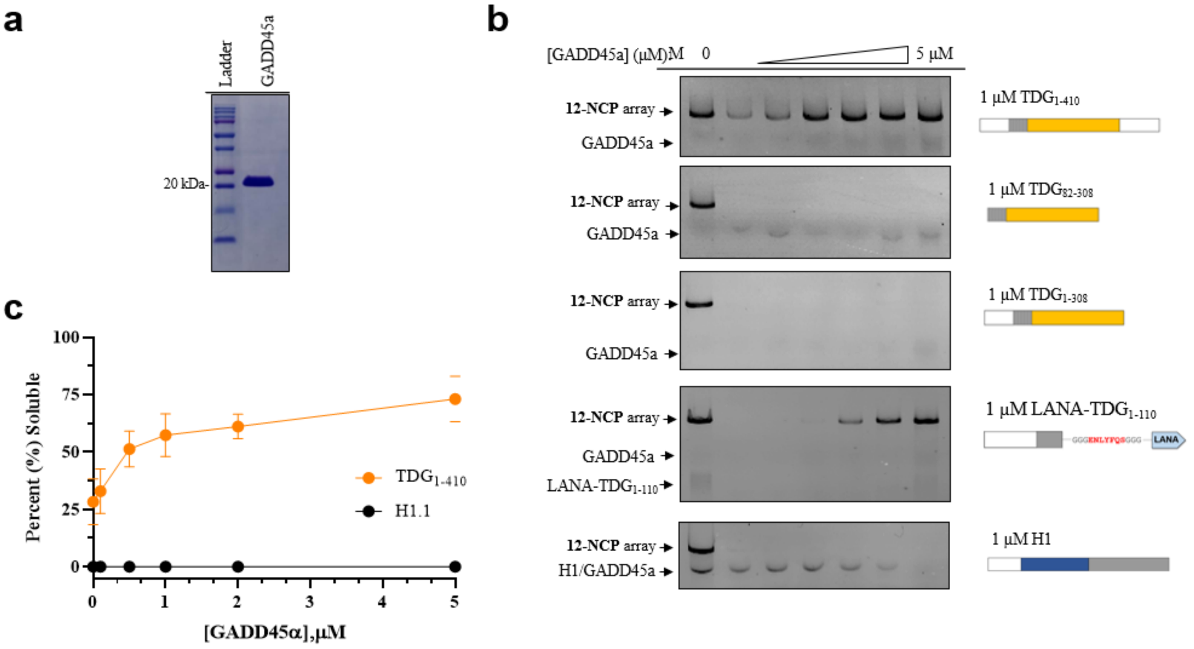

Figure S11. GADD45a reverses insoluble TDG-chromatin oligomers. (a) Denaturing SDS PAGE (15%) analysis of recombinant human GADD45a protein stained with Coomassie Brilliant Blue. Ladder is the Bio-Rad Precision Plus Pre-stained ladder (cat. no. 1610374). (b) Representative gel images showing the soluble fraction from experiments in which **12-NCP** arrays were first aggregated by the indicated TDG variant or H1 (to the right) and subsequently exposed to increased concentrations of GADD45a. M: **12-NCP** array marker. (c) Solubility plot comparing the effects of GADD45a on full-length TDG- and H1-condensed chromatin; triplicate representation of the experiments depicted in panel (b). Error bars represent standard deviation from at least three independent experiments.

Figure S12

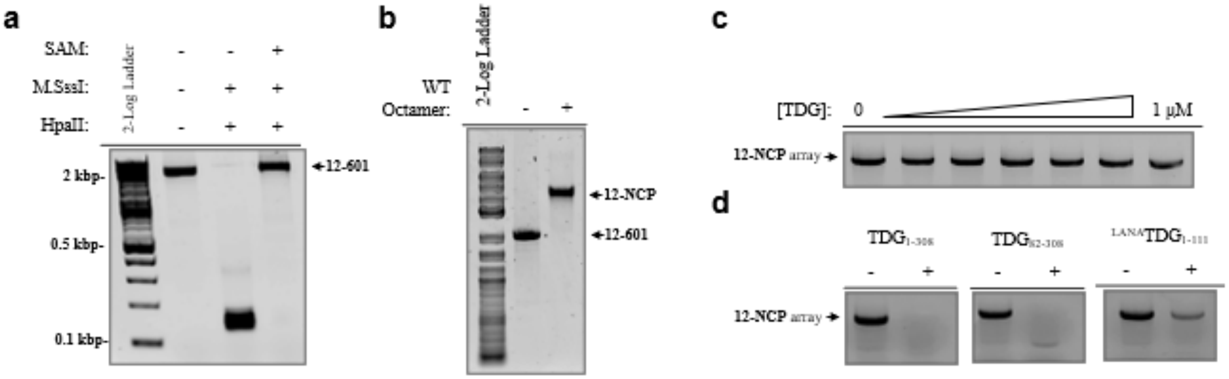

Figure S12. Chromatin condensation by TDG is inhibited by DNA methylation. (a) Agarose gel electrophoresis (0.7%) analysis of CpG hypermethylated **12-601** DNA digested by HpaII. DNA treated with M.SssI and SAM was resistant to HpaII cleavage, while control reactions (lacking SAM) were digested completely by HpaII, confirming complete CpG methylation. (b) Agarose gel analysis (0.6%) of chromatin reconstitution reactions using the HpaII-resistant (hypermethylated) DNA depicted in part (a). (c,d) Representative gel images showing the soluble fraction of methylated **12-NCP** arrays following treatment with increasing concentrations of full-length TDG (c) or 1 μM truncated TDG variants (d).

Figure S13

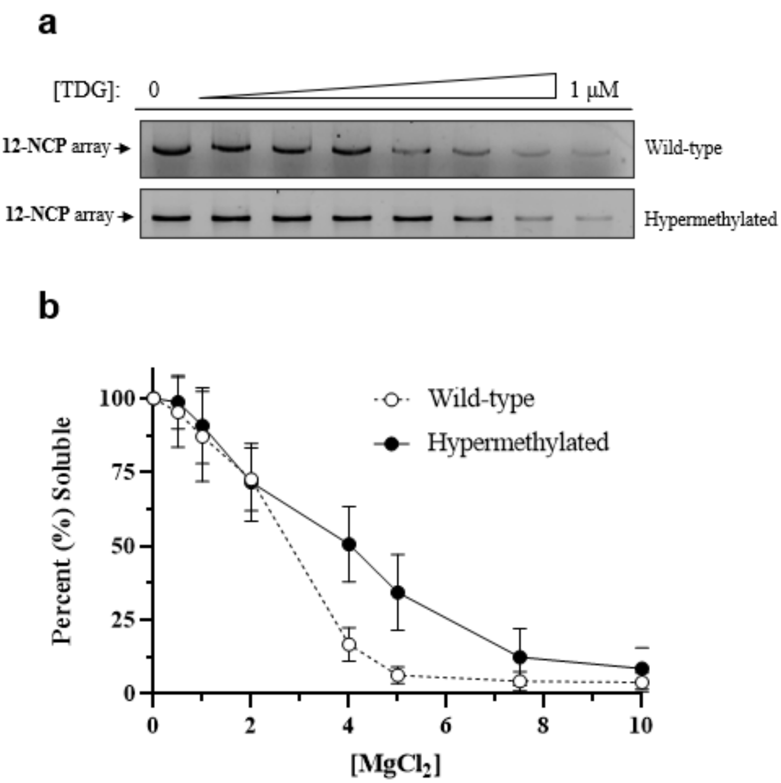

Figure S13. DNA methylation modestly impedes Mg^{2+}-induced condensation of 12-mer nucleosome arrays. (a) Representative gel images showing the soluble fraction of wild-type and hypermethylated **12-NCP** arrays following treatment with the indicated concentrations of full-length TDG. (b) Solubility plot of the experiments depicted in (a).

Figure S14

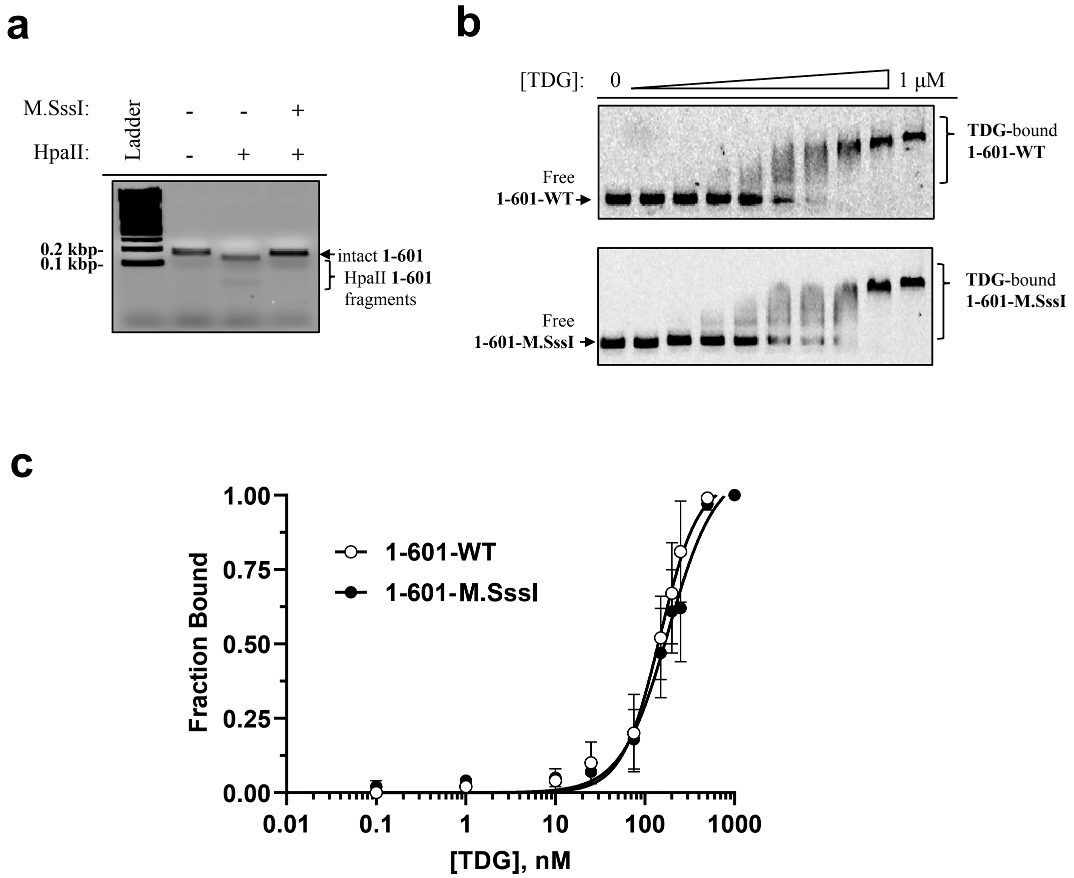

Figure S14. TDG binds 601 DNA irrespective of DNA methylation. (a) Agarose gel image confirming the resistance of M.SssI-treated **1-601** (207 bp) to HpaII cleavage. (b) Representative agarose gel (0.8%) gel images of full-length TDG with unmethylated (**1-601**) and M.SssI-treated (**1-601-M.SssI**) 601 DNA. (c) Saturation plots of triplicate binding experiments depicted in part (b). Binding was carried out in buffer NB supplemented with 5% glycerol as described in the methods.

Figure S15

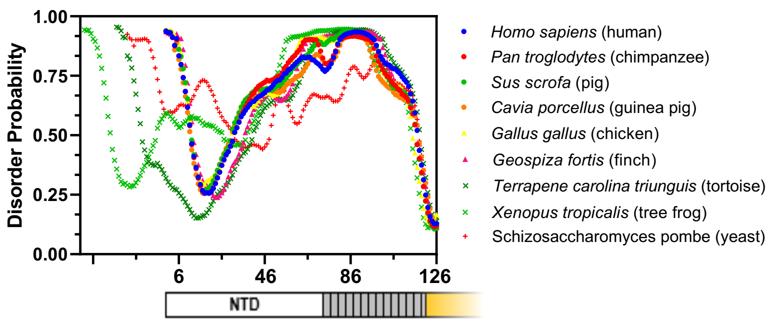

Organism	Mean Disorder Probability	Amino Acid Abundance (NTD)				Sequence Similarity	
	**NTD**	**K/R**	**P**	**A/G**	**Q/N/S**	**NTD**	**Overall**
6996-*Homo sapiens*	67.6%	16.8%	10.4%	13.6%	17.6%	-	-
452188-*Pan troglodytes*	67.7%	17.6%	11.2%	13.6%	17.6%	99.2%	99.3%
100155184-*Sus scrofa*	70.5%	17.5%	10.8%	14.2%	16.7%	85.8%	91.9%
100724604-*Cavia porcellus*	65.1%	18.1%	11.8%	22.8%	11.8%	78.6%	82.0%
395510-*Gallus gallus*	68.8%	20.0%	8.8%	15.2%	9.6%	55.7%	75.7%
102035597-*Geospiza fortis*	66.8%	20.3%	9.4%	14.8%	12.5%	55.9%	75.2%
112106684-*Terrapene Carolina triunguis*	61.5%	15.7%	10.7%	14.3%	15.0%	65.1%	80.6%
448432-*Xenopus tropicalis*	62.1%	11.2%	9.4%	14.1%	18.2%	43.8%	61.3%
2539432-*Schizosaccaromyces pombe*	60.7%	18.3%	2.0%	9.2%	20.9%	N/A	36.6%

Figure S15. Disorder probability and residue content for select vertebrate and yeast TDG homologs. The disorder probability for TDG homologs were computed using Protein Disorder Prediction System (PrDOS)[9] and aligned using the calculated disorder probabilities from the catalytic domain. Mean disorder probability was calculated from the averaged predicted values for the TDG N-terminal domain, which considered every amino acid upstream of the PrDOS minima bordering the catalytic domain from each TDG species; this site corresponds to residue D126 in *h*TDG. Sequence similarities were determined for the same NTD residues, and the full-length proteins, with respect to *h*TDG via NCBI Blast protein alignment tool.

S3. SUPPLEMENTARY TABLES

Table S1.

Protein	Chromatin Condensation	5mC-sensitive	Reversibility	
			DNA	GADD45a
TDG	++	Y	Y	Y
H1.1	+++	N	Y	N
TDG_{1-308}	+++	N	N	N
TDG_{82-308}	+++	N	N	N
$TDG_{111-308}$	+	Y	Y	Y
$TDG_{308-410}$	-	ND	ND	ND
LANA-TDG_{1-110}	++	Y	ND	Y
LANA-$TDG_{309-410}$	-	ND	ND	ND

Table S1. Summary of TDG-mediated chromatin condensation efficiency and reversibility results. Chromatin condensation efficiency was classified based A_{50} values (i.e. the concentration that yield 50% precipitation): an $A_{50} \leq 250$ nM is depicted as +++, an A_{50} between 250- 500 nM is depicted as ++, an A_{50} > 500 nM is depicted as +, and a lack of detectable precipitation is represented by a (-). Sensitivity to cytosine methylation (5mC) and reversibility is indicated as yes (Y) or no (N).

S4. SUPPLEMENTARY REFERENCES

(1) Deckard, C.D. III, Banerjee, D.R.; Sczepanski, J.T. Chromatin Structure and the Pioneering Transcription Factor FOXA1 Regulate TDG-Mediated Removal of 5-Formylcytosine from DNA. *J. Am. Chem. Soc.* **2019**, *141*, 11410–11414.

(2) Poirier, M.; Oh, E.; Tims, H.; Widom, J. Dynamics and Function of Compact Nucleosome Arrays. *Nat. Struct. Mol. Biol.* **2009**, *16* (9), 938–944. https://doi.org/10.1038/nsmb.1650.

(3) Banerjee, D. R.; Deckard, C. E.; Elinski, M. B.; Buzbee, M. L.; Wang, W. W.; Batteas, J. D.; Sczepanski, J. T. Plug-and-Play Approach for Preparing Chromatin Containing Site-Specific DNA Modifications: The Influence of Chromatin Structure on Base Excision Repair. *J. Am. Chem. Soc.* **2018**, *140* (26), 8260–8267. https://doi.org/10.1021/jacs.8b04063.

(4) Hieb, A. R.; D'Arcy, S.; Kramer, M. A.; White, A. E.; Luger, K. Fluorescence Strategies for High-Throughput Quantification of Protein Interactions. *Nucleic Acids Res.* **2011**, *40* (5), 1–13. https://doi.org/10.1093/nar/gkr1045.

(5) Gansen, A.; Felekyan, S.; Kühnemuth, R.; Lehmann, K.; Tóth, K.; Seidel, C. A. M.; Langowski, J. High Precision FRET Studies Reveal Reversible Transitions in Nucleosomes between Microseconds and Minutes. *Nat. Commun.* **2018**, *9* (1), 4628. https://doi.org/10.1038/s41467-018-06758-1.

(6) Gansen, A.; Hieb, A. R.; Böhm, V.; Tóth, K.; Langowski, J. Closing the Gap between Single Molecule and Bulk FRET Analysis of Nucleosomes. *PLOS ONE* **2013**, *8* (4), e57018. https://doi.org/10.1371/journal.pone.0057018.

(7) Iqbal, A.; Arslan, S.; Okumus, B.; Wilson, T. J.; Giraud, G.; Norman, D. G.; Ha, T.; Lilley, D. M. J. Orientation Dependence in Fluorescent Energy Transfer between Cy3 and Cy5 Terminally Attached to Double-Stranded Nucleic Acids. *Proc. Natl. Acad. Sci.* **2008**, *105* (32), 11176–11181. https://doi.org/10.1073/pnas.0801707105.

(8) Cunningham, P. D.; Khachatrian, A.; Buckhout-White, S.; Deschamps, J. R.; Goldman, E. R.; Medintz, I. L.; Melinger, J. S. Resonance Energy Transfer in DNA Duplexes Labeled with Localized Dyes. *J. Phys. Chem. B* **2014**, *118* (50), 14555–14565. https://doi.org/10.1021/jp5065006.

(9) Ishida, T.; Kinoshita, K. PrDOS: Prediction of Disordered Protein Regions from Amino Acid Sequence. *Nucleic Acids Res.* **2007**, *35* (suppl_2), W460–W464. https://doi.org/10.1093/nar/gkm363.

(10) Maeshima, K.; Rogge, R.; Tamura, S.; Joti, Y.; Hikima, T.; Szerlong, H.; Krause, C.; Herman, J.; Seidel, E.; DeLuca, J.; Ishikawa, T.; Hansen, J. C. Nucleosomal Arrays Self-Assemble into Supramolecular Globular Structures Lacking 30-Nm Fibers. *EMBO J.* **2016**, *35* (10), 1115–1132. https://doi.org/10.15252/embj.201592660.
